# Characterization of the mammalian miRNA turnover landscape

**DOI:** 10.1093/nar/gkv057

**Published:** 2015-02-04

**Authors:** Yanwen Guo, Jun Liu, Sarah J. Elfenbein, Yinghong Ma, Mei Zhong, Caihong Qiu, Ye Ding, Jun Lu

**Affiliations:** 1 Department of Genetics, Yale University School of Medicine, New Haven, CT 06510, USA; 2 Yale Stem Cell Center, Yale Cancer Center, New Haven, CT 06520, USA; 3 Graduate Program in Biological and Biomedical Sciences, Yale University, New Haven, CT 06510, USA; 4 Computational Biology and Bioinformatics Program, Yale University School of Medicine, New Haven, CT 06520, USA; 5 Wadsworth Center, New York State Department of Health, Albany, NY 12208, USA; 6 Yale Center for RNA Science and Medicine, New Haven, CT 06520, USA

## Abstract

Steady state cellular microRNA (miRNA) levels represent the balance between miRNA biogenesis and turnover. The kinetics and sequence determinants of mammalian miRNA turnover during and after miRNA maturation are not fully understood. Through a large-scale study on mammalian miRNA turnover, we report the co-existence of multiple cellular miRNA pools with distinct turnover kinetics and biogenesis properties and reveal previously unrecognized sequence features for fast turnover miRNAs. We measured miRNA turnover rates in eight mammalian cell types with a combination of expression profiling and deep sequencing. While most miRNAs are stable, a subset of miRNAs, mostly miRNA*s, turnovers quickly, many of which display a two-step turnover kinetics. Moreover, different sequence isoforms of the same miRNA can possess vastly different turnover rates. Fast turnover miRNA isoforms are enriched for 5′ nucleotide bias against Argonaute-(AGO)-loading, but also additional 3′ and central sequence features. Modeling based on two fast turnover miRNA*s miR-222-5p and miR-125b-1-3p, we unexpectedly found that while both miRNA*s are associated with AGO, they strongly differ in HSP90 association and sensitivity to HSP90 inhibition. Our data characterize the landscape of genome-wide miRNA turnover in cultured mammalian cells and reveal differential HSP90 requirements for different miRNA*s. Our findings also implicate rules for designing stable small RNAs, such as siRNAs.

## INTRODUCTION

MiRNAs are essential players in the post-transcription layer of gene expression regulation (1). The patterns of mature miRNA expression are highly dynamic during development and differentiation, whereas dysregulation of miRNA expression is observed frequently in human diseases (2,3). Mature miRNA levels can be controlled by transcriptional changes and miRNA processing (4). The mechanism that has been less explored is miRNA turnover.

miRNA turnover and biogenesis can be closely related. miRNA genes are transcribed into hairpin-containing precursors, which are processed by Drosha and Dicer, resulting in ∼22-nt double-stranded RNA duplex (reviewed in (5)). This duplex is loaded onto AGO proteins, requiring molecular chaperons. Particularly, the current model specifies that HSP90 and its ATPase activity are required for AGO loading of miRNA duplexes (6–11). However, it is not known whether all miRNAs are equally dependent on this pathway. In addition, while biochemical assays with synthetic miRNA or siRNA duplexes demonstrate a role of HSP90 in the AGO-loading process, there has been a lack of direct evidence for endogenous cellular miRNA(s) in complex with HSP90/AGO. After miRNA duplex binding to AGO/HSP90 complex, only one of the strands is successfully loaded into AGO-containing RNA-induced silencing complex (RISC) to mediate gene silencing, whereas the other strand is discarded and often thought to be degraded right away. However, the *in vivo* kinetics of these processes in mammalian cells is not clear.

For most miRNAs within cells, one strand of the miRNA duplex has a higher chance to load into RISC complex and of higher abundance at steady state, even though the two strands are equimolarly produced from the same hairpin. The less abundant strand has historically been referred to as miRNA star (miRNA*), some of which can be loaded into RISC (12) and impart wide-spread regulation of gene expression (13). The rules that govern miRNA strand imbalance in mammalian cells are believed to be mostly determined by the relative thermodynamic stability of the two ends of miRNA duplex (14). In *Drosophila*, first nucleotide sequence bias has also been shown to regulate RISC loading efficiency (12,15,16). Consistent with this, human AGO2 was shown to have structural preference for the first-base of U and A and less so for G or C (17). Besides these, it is not clear whether other sequence motifs are associated with differential preference of miRNA strands in the RISC-loading process.

It is widely believed that mammalian miRNAs have high stability (18–20). Evidence supporting this notion, however, was often obtained in single-cell line experiments, anecdotal, or in a whole organism as a mixture (e.g. (21–24)). Although AGO loading can provide protection of miRNAs from degradation (19), AGO levels vary in different cell types and other stability regulatory mechanisms exist, raising the question whether different turnover behavior is present in previously uncharacterized cell backgrounds. On the other hand, a small number of specific miRNAs have been reported to possess faster turnover kinetics, often with half-lives of ∼3–8 h (reviewed in (19,25)). While the half-lives of some quick-turnover miRNA*s are estimated to be ∼3.5 h in worms (23), the turnover behavior and half-lives of miRNA*s in mammalian cells are largely unknown.

To better understand the landscape of mammalian miRNA turnover, associated miRNA sequence features and the biogenesis process, we undertook a comprehensive approach to measure miRNA half-lives in eight mammalian cell types by a combination of both miRNA profiling and next-generation sequencing. While most miRNAs are stable, a subset of miRNAs, mostly miRNA*s, turnovers quickly. We found that ∼50% of fast turnover miRNAs display a two-step turnover kinetics, with the first fast turnover phase having a half-life of ∼1 h. Our data also indicate that multiple isoforms of the same miRNA can co-exist in cells but with distinct turnover kinetics. Using the fast turnover miR-222-5p (miR-222*) and miR-125b-1-3p (miR-125b-1*) as models, we demonstrate that these two miRNAs are associated with AGO; however, they differ drastically with respect to HSP90 association and HSP90 dependence. Our data provide evidence for endogenous miRNAs in complex with HSP90 and demonstrate differential sensitivity of miRNA*s toward HSP90 inhibition. Furthermore, we report several previously unknown sequence features and motifs associated with fast turnover miRNAs, including those at the 3′ end and those in the middle of miRNA sequences.

## MATERIALS AND METHODS

### Cell culture

HeLa, PC-3, IMR-90, mouse embryonic fibroblast (MEF) and MCF-7 cells were cultured in Dulbecco's modified Eagle's medium (DMEM) supplemented with 10% fetal bovine serum (FBS) and 1% penicillin-streptomycin. HDMYZ, K562 and Raji cells were cultured in RPMI-1640 medium with 10% FBS and 1% Penicillin-Streptomycin. mESCs were cultured on MEF feeders in DMEM media supplemented with 15% FBS (Hyclone), 1% MEM non-essential amino acids, 1% penicillin-streptomycin, 0.8% 2-mercaptoethanol and 1000-U/ml ESGRO (Millipore). For harvesting mouse embryonic stem cells (mESCs) samples, cells were de-MEFed by trypsinizing and plating on Gelatin-coated (Millipore) plates for 40 min to deplete MEF.

Human embryonic stem cell research was performed on a protocol approved by the hESC Oversight Committee at Yale University. H1 hESCs (WA01, WiCell) were maintained in an undifferentiated state by culturing on Matrigel-coated plates (BD Biosciences), with DMEM/F12 medium supplemented with 1% MEM-non-essential amino acids, 1-mM L-glutamine, 1% penicillin-streptomycin, 50-ng/ml bFGF (Millipore), 1X N2 and 1X B27 supplements (Invitrogen) and with a daily medium change.

Differentiation of hESCs into neuronal progenitor cells was performed following a published method (26) with modifications. Embryoid bodies were generated by detaching undifferentiated H1 colonies from plates with dispase (Stem Cell Technology) and put on extra-low attachment plates (Corning) with DMEM/F12, supplemented with 2-mM L-glutamine, 1% P/S, 1X B27 supplements, 250-ng/ml Noggin (PeproTech) and 20-ng/ml bFGF. The medium was changed every 2 days for a total of 4 weeks with the last week medium eliminating Noggin. All cell culture reagents were from Invitrogen unless noted otherwise.

### Actinomycin D, DRB and 17-DMAG treatment and sample collection

Cells were plated in six-well plates at ∼200 000 cells/well. A media change was performed 1 day after plating. Actinomycin D (ActD; Sigma) and DRB (Sigma) treatment were started around 2 days after plating for 0, 2, 4, 6, 12 and 24 h. To understand the roles of HSP90 inhibition, HDMYZ cells were treated with 1 μM of 17-DMAG (Sigma) for 0, 4, 8, 12 and 24 h. In addition, for concentration titration, HDMYZ cells were treated with 0.008, 0.04, 0.2, 1, 5 and 25 μM of either 17-DMAG or 17-AAG (Sigma) for 4 h. Experiments for each time point were performed with biological duplicate (ActD treatment) or triplicate (DRB and 17-DMAG treatment). The concentration of ActD and DRB for different cell lines was titrated so that cells were mostly alive after 24 h of treatment. Effectiveness of treatment was verified by analyzing c-myc RNA; see Supplementary Table S1 for ActD and DRB concentrations. After treatment at indicated time points, cells were washed with phosphate buffered saline (PBS) once and lysed in TriZol reagent immediately (Invitrogen). RNA extraction was performed following the manufacturer's protocol.

### miRNA expression profiling

Cells are washed once with cold PBS (Invitrogen) and lysed with TRIzol (Invitrogen). Total RNA extraction was performed following the manufacturer's protocol. miRNA profiling was performed using a bead-based method following a published protocol (27). Of note, we spiked three synthetic small RNA molecules with 5′ phosphate and 3′OH into total RNA at a defined ratio relative to total RNA quantity, with details of sequences and procedures described previously (28,27).

To normalize miRNA expression profiling data, two methods were used. To derive quantification of total miRNA amount, we normalized profiling data using the three synthetic small RNAs, using a published procedure (27), so that the normalized data reflect miRNA expression relatively to total RNA quantity. We then added the intensity values for all mature miRNA probes to reflect total miRNA amount. After we found that total miRNA amount was largely stable, we used a second normalization process on the raw profiling data, assuming that total miRNA amount is constant between samples. This procedure was described previously (28).

### Next-generation sequencing

HDMYZ cells were treated with 4-μg/ml ActD for 0, 1, 4 and 12 h. Small RNAs of 15–40 bases were gel-purified from 5-μg total RNA with 10% acrylamide gel (American Bioanalytical AB13021). Purified small RNA was subjected to library preparation, similar to Illumina protocol with modification. Briefly, pre-adenylated primer was made following a published protocol (29) using /5Phos/TGGAATTCTCGGGTGCCAAGG/3ddC/. Small RNA was ligated to the pre-adenylated primer with truncated T4 RNA ligase 2 (NEB M0242S). The 35–55 bases product was purified with 10% acrylamide gel and further went through a 5′ ligation reaction with primer: dGdTdTdCdAdGdAdGdTdTdCdTdAdCdA GUCCGACGAUC (Dharmacon) with T4 RNA ligase 1 (Fermentas EL0021). The 60–80 bases product was purified with 10% acrylamide gel and reverse transcribed with M-MLV reverse transcriptase (RT) (Invitrogen 28025–013) using primer: GCCTTGGCACCCGAGAATTCCA. The RT product was polymerase chain reaction (PCR) amplified for 18 cycles with Phusion DNA polymerase (NEB M0530) using a universal primer: AATGATACGGCGACCACCGAGATCTACACGTT-CAGAGTTCTACAGTCCGA and a specific primer for each sample (listed in Supplementary Table S4). 130–150-nt small RNA libraries were purified with 8% acrylamide gel. Barcoded small RNA libraries were sequenced on a HiSeq2000 (Illumina).

### Analysis of next-generation sequencing data

Sequence reads were processed by the miRDeep2 package (30), with the following modifications. First, reads for the same library obtained on different sequencing runs were combined as a first step. Second, we did not restrict the size of small RNAs during adaptor removal. Third, we used miRBase release 18 (31,32) for mapping the reads. Fourth, for quantifying miRNA and isoform frequency, we limited reads to more than or equal to 15 bases in length with zero mismatch during mapping. In addition, to examine 3′ non-templated nucleotide additions, we remapped the data to miRBase, allowing up to two bases of mismatches. We then filtered the mapping results to allow only mismatches to the 3′ end of miRNAs, with either single base or two-base mismatches.

The number of reads that were mapped to known miRNAs (with zero mismatch) was used to normalize read frequencies for each miRNA or each miRNA isoform, assuming that total miRNA reads (mapped to mature miRNAs) were constant between samples. For quantification purposes, we only considered miRNAs or isoforms that had mean frequency > = 5 × 10e-6 in samples without ActD treatment (i.e. at 0 h). These miRNAs or isoforms were referred to as reliably quantifiable.

When comparing miRNA turnover within star and non-star miRNAs, we annotated star and non-star strands based on the sequencing data from HDMYZ cells. Specifically, for miRNA precursors that have two mature miRNAs in miRBase, we compared the mean frequency for the two strands for samples at 0 h, and the more abundant strand was annotated as non-star, whereas the less abundant strand was annotated as star. Of note, annotations based on HDMYZ data agreed with miRBase annotation in most cases (Supplementary Table S2). For those precursors with only one annotated mature miRNA, such miRNA was taken as the non-star sequence.

To analyze miRNA isoforms, we used custom Perl scripts to quantify sequence-level Bowtie (33) mapping results from miRDeep2 package (30). Of note, we can observe sequences corresponding to loop regions of miRNA hairpin, which reflect Dicer processing byproducts. To analyze associated sequence features, we focused on 1036 sequence isoforms that both mapped to mature miRNA regions and passed the reliably quantifiable threshold. For the fast turnover group, we assign isoforms that had <50% or 25% abundance after 12 h of ActD treatment and had nominal *P*-value <0.05 for any of the following comparisons: between 0 and 4 h, between 0 and 12 h, or 0 versus 4 and 12 h. For the slow turnover group, we assigned those isoforms that remain at least 75% after 12 h of ActD treatment and do not show significant changes according to the above criteria. The nucleotide composition at each nucleotide position for fast and slow turnover miRNA isoforms was computed. Permutation test was performed by randomly selecting the same number of isoforms as the fast turnover group from the pool of both fast and slow turnover isoforms, with 10 000 iterations. Permutation *P*-values were calculated by the number of iterations leading to more extreme frequency of a given nucleotide at a specific position over the total number of iterations.

### RNA immunoprecipitation and western blot

Immunoprecipitation assay was performed following a published protocol (34) with the following modifications. HDMYZ cells were lysed for immunoprecipitation. Lysate was collected for input as well as for immunoprecipitation using 140–400 μg (based on protein content) of protein lysate. Immunoprecipitation was performed with 10 μg of pan-AGO antibody (MABE56, Millipore), Ago2 antibody (015-22031, Wako Chemicals), Hsp90 antibody (sc-13119, Santa Cruz) and IgG antibody (12–371, Millipore) at 4°C overnight after pulling down with 30 μl of Protein A/G-conjugated magnetic beads (Pierce). For experiments examining ActD treatment, HDMYZ cells were treated with 4-μg/ml ActD or the same volume of DMSO for 4 h before harvesting. After immunoprecipitation, samples were collected in TRIzol LS reagent (Invitrogen) for RNA extraction. RNA was co-precipitated with 15 μg of glycogen. Calculations of immunoprecipitation (IP) experiments were based on fractions relative to the input material (see quantitative RT-PCR (qRT-PCR) for details).

For quality control of the immunoprecipitation assay, western blot was performed as described before (35). Protein samples were collected as input, IP and flow-through samples. Input samples refer to the initial cell lysate before IP, IP samples contain proteins bound by beads after immunoprecipitation and flow-through samples correspond to proteins not bound by the beads during the initial bead-binding step. Protein samples were added into sodium dodecyl sulphate loading buffer and analyzed using 10% polyacrylamide gels. Loading of samples followed the corresponding fractions of samples. For example, IP samples from the same fraction of input material were labeled as 1x Input.

### Statistical analysis

Student's *t*-test was used for statistical analyses unless noted otherwise. When analyzing base distribution, the numbers of a given nucleotide at a specific position were calculated from fast turnover miRNA isoforms and slow turnover miRNA isoforms, in comparison to total slow and fast turnover miRNA isoform numbers. *P*-values were calculated using Fisher's exact test. Note that we also calculated *P*-values using permutation test, which were similar to those obtained by Fisher's exact tests.

### Additional methods

Additional methods, including constructs, qRT-PCR, measurement of miRNA turnover with an inducible system, measurement of mutant miR-222 turnover and measurement of miR-222 secretion, can be found in the Supplemental Materials.

### Data access

The miRNA profiling data were deposited in GEO (GSE46877). The HDMYZ next-generation sequencing data were deposited in GEO (GSE60036). The MCF7 next-generation sequencing data (for mutant miR-222) were deposited in GEO (GSE64363).

## RESULTS

### Using transcriptional inhibitors to quantify miRNA turnover rates in multiple mammalian cell types

**Figure 1.**
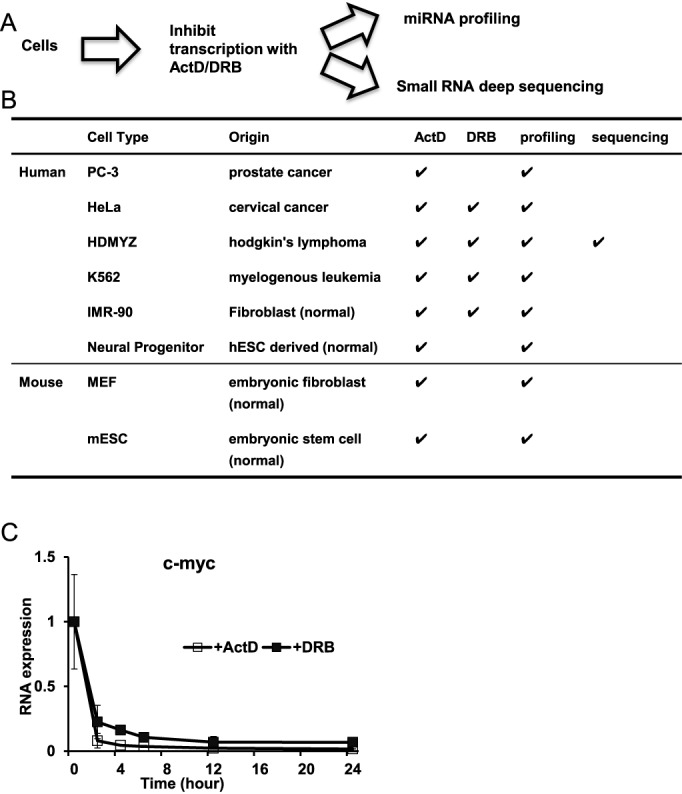
Measuring miRNA turnover kinetics with transcription inhibitors. (**A**) Schematic of experimental design to measure cellular miRNA turnover kinetics. Cultured mammalian cells were treated with transcription inhibitors Actinomycin D (ActD) or 5,6-Dichlorobenzimidazole 1-β-D-ribofuranoside (DRB). Total RNA samples collected in a time course experiment were measured by miRNA profiling and/or small RNA deep sequencing. (**B**) Different cell types, corresponding drug treatment and miRNA measurement approaches are listed, with check marks indicating performed experiments. (**C**) Levels of c-myc mRNA post ActD and DRB treatment for the indicated time points were measured by qRT-PCR. Data from HDMYZ cells are shown as an example. Data were normalized to 0-h time point. *N* = 2 for ActD-treated samples. *N* = 3 for DRB-treated samples. Error bars stand for standard deviations with *N* = 3, or average deviations from median with *N* = 2.

To investigate the landscape of mature miRNA turnover rates in cultured mammalian cells, we first carefully selected eight cultured cell types to represent a large spectrum of mammalian cells, as it is possible that different cell types might have distinct mechanisms of miRNA turnover and/or substantially different AGO levels. Our selection covers human and mouse origins, as well as cancerous and normal cell states (Figure 1A and B), including human cancer cell lines (prostate cancer PC-3, cervical cancer HeLa, lymphoma HDMYZ and chronic myeloid leukemia K562), normal human cell types (lung fibroblast IMR-90 and neural progenitors derived from human embryonic stem cell line H1) and normal murine cell types (primary MEFs and mouse embryonic stem cells).

To measure miRNA turnover rates, we resorted to a well-established approach to inhibit transcription with chemical inhibitor(s), followed by quantification of the rates of miRNA abundance decay (Figure 1A). We used ActD that inhibits transcription from all three mammalian RNA polymerases and DRB (5,6-Dichlorobenzimidazole 1-β-D-ribofuranoside), which elicits a different mechanism of transcriptional inhibition by inhibiting RNA polymerase II activity. To confirm effective transcriptional inhibition, we measured the turnover of c-myc, a fast turnover messenger RNA (36), in all samples. Indeed, c-myc was rapidly downregulated within 2 h in each cell type, indicating efficient transcriptional inhibition (Figure 1C and Supplementary Figure S1).

To quantify miRNA turnover, we took two parallel and complementary approaches. First, we profiled miRNA expression on a bead-based detection platform that has high specificity and good sensitivity (27). This approach, due to its inexpensive nature, allowed us to quantify 582 mature human and mouse miRNAs in all of the eight cell types, with biological triplicate or duplicate for six time points (at 0, 2, 4, 6, 12 and 24 h). In total, we successfully produced 208 expression profiles, resulting in 121 056 miRNA measurements.

Second, in a complementary effort, we measured miRNA turnover in a specific cell line HDMYZ using next-generation sequencing, so that we could gain more information on lower abundance miRNA species and miRNA isoforms. HDMYZ cells were treated with ActD and collected at 0, 1, 4 and 12 h post-treatment with biological triplicate. Small RNAs were then gel-purified and sequenced using the Illumina Hi-Seq platform. In total, 41 million reads were obtained that could be mapped to known human miRNA precursors with perfect match. Variations among biological replicates are relatively small (Supplementary Figure S2A; 90% of miRNAs having standard deviation over mean ratio of less than 0.4, when miRNAs are above detection threshold), supporting that the data are of good quality. With both miRNA profiling and sequencing data, we went on to examine the landscape of miRNA turnover rates.

### The majority of detectable miRNAs are stable, whereas a subset of miRNAs displays fast turnover rates

**Figure 2.**
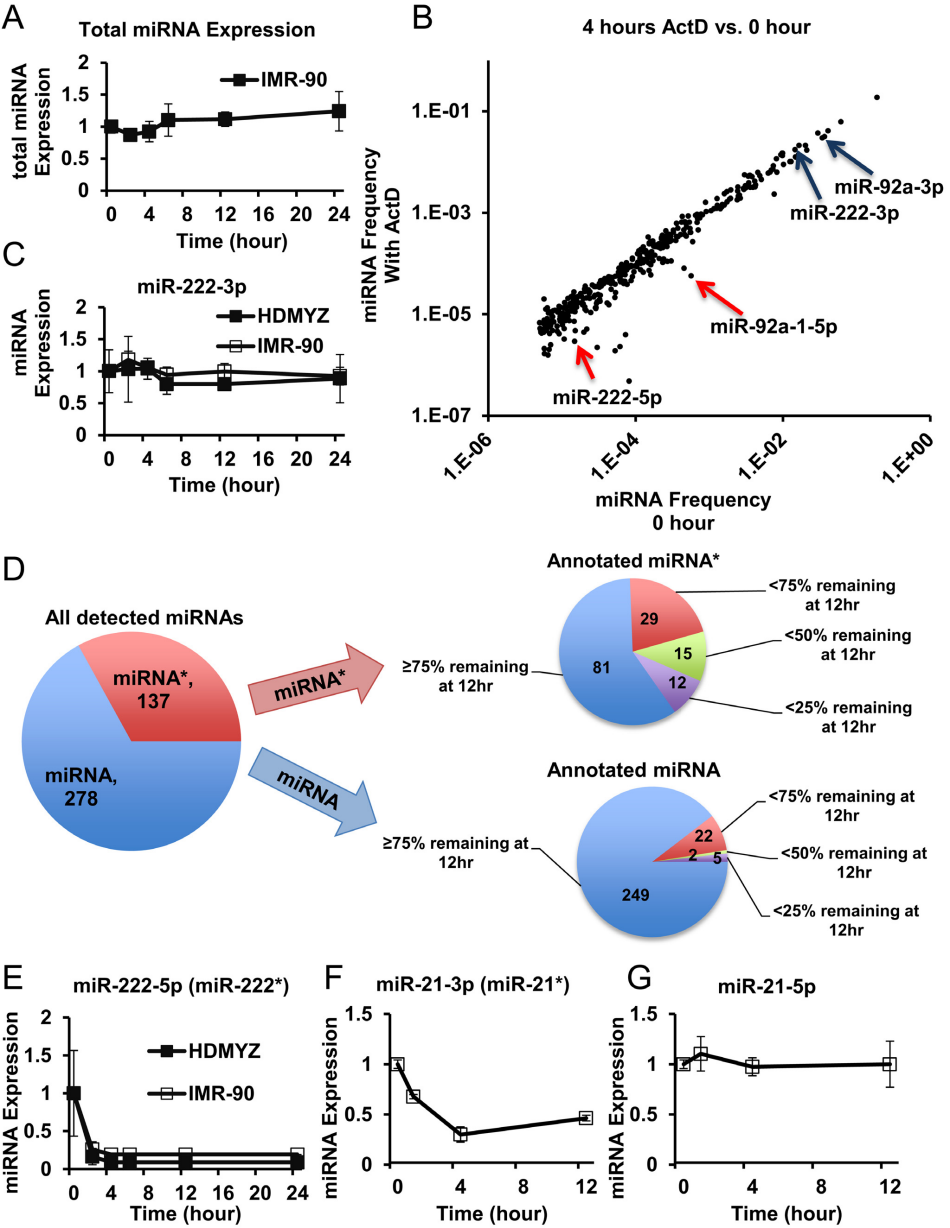
miRNA profiling and deep sequencing reveal stable and fast turnover miRNAs. (**A**) Total miRNA profiling intensities were measured (see the Materials and Methods section) in cells after ActD treatment at indicated time points, and normalized against total RNA amount. Data from IMR-90 cells are shown as an example. *N* = 2. (**B**) Dot plots of normalized frequency of miRNA sequencing reads are shown, for comparison between HDMYZ cells without treatment and those treated with ActD for 4 h. Data represent the average from biological triplicates. Each dot represents one annotated mature miRNA. Two fast turnover miRNAs (miR-222-5p and miR-92a-1-5p) are indicated with arrows, their slow turnover counterparts (miR-222-3p and miR-92a-3p) are also indicated with arrows. (**C**) miR-222-3p turnover kinetics measured by miRNA profiling in HDMYZ and IMR-90 cells at indicated time points post-ActD treatment. *N* = 2. (**D**) Statistics of the turnover of all detectable miRNAs in HDMYZ cells. A total of 415 miRNAs were considered reliably quantifiable (see the Materials and Methods section). The more abundant strand from an miRNA hairpin is annotated as ‘miRNA’, with the less abundant strand as ‘miRNA*’. Numbers of miRNAs with indicated turnover kinetics are labeled in the pie charts. (**E, F, G**) Examples of differential miRNA turnover kinetics are shown. (E) Levels of miR-222-5p were measured by miRNA profiling in HDMYZ and IMR-90 cells at indicated time points post-ActD treatment. *N* = 2. (F, G) Levels of miR-21-3p and miR-21-5p were measured by deep sequencing after ActD treatment in HDMYZ cells. *N* = 3. Error bars stand for standard deviations with *N* = 3, or average deviations from median with *N* = 2.

To determine the landscape of miRNA turnover rates, we first examined the turnover kinetics of total miRNA abundance. To achieve this, we utilized artificial miRNA mimics (non-mammalian sequence) that were spiked into RNA samples before profiling (see the Materials and Methods section (27)). Thus, profiling data normalized based on signals from spiked-in miRNA-mimics effectively reflect miRNA abundance per total RNA quantity. We next examined the sum of all miRNA signals to reflect total miRNA abundance. In all examined cell types, the total miRNA abundance showed slow turnover rates (Figure 2A and Supplementary Figure S3A and B), with no or minor decrease within 24 h. In some cases, the total miRNA abundance displayed an increase with time. This phenomenon is possible because ribosomal RNAs (the major component of total RNA) have half-lives of ∼18 h, and miRNA turnover could be slower than this rate. The data above support a slow turnover rate of total miRNAs in all cell types examined.

The slow rate of total miRNA turnover allowed us to normalize both miRNA expression profiling and sequencing data assuming total miRNA expression did not change across time points as a close approximation. Indeed, most miRNAs (post-normalization) are lined up close to the diagonal line when comparing sequencing samples treated with ActD versus 0-h samples (Figure 2B and Supplementary Figure S2B), indicating that this normalization assumption is reasonable.

Examining the turnover kinetics of specific miRNAs within the miRNA expression profiling data revealed that the vast majority of miRNAs had half-lives longer than 18 h. For example, miR-222-3p showed largely steady levels in all cell types that express this miRNA (Figure 2C and Supplementary Figure S3C). We validated the profiling results with qRT-PCR on miR-222-3p in HDMYZ cells (Supplementary Figure S3D). Consistent with the profiling data, sequencing results indicate that 330 of the 415 detectable miRNAs showed at least 75% remaining levels after 12 h of ActD treatment (Figure 2D and Supplementary Table S2). The data above indicate that most detectable miRNAs in all of the eight cell types have long half-lives. This result not only is consistent with the notion of high miRNA stability in the field (18–20) but also demonstrates that this phenomenon is widespread across many different cell types and across most miRNAs.

While most detectable miRNAs are stable, we also noticed that a small subset of miRNAs demonstrated fast or ultrafast turnover rates. In the sequencing data from HDMYZ cells, when comparing to 0-h samples, the levels of a group of miRNAs were substantially decreased at 4- and 12-h ActD time points (dots below diagonal in Figure 2B and Supplementary Figure S2B). Among a total of 415 miRNAs that passed our detection threshold, 17 (4%) miRNAs had <25% remaining after 12 h of ActD treatment, whereas 34 (8%) had <50% remaining (Figure 2D and Supplementary Table S2). Examples include miR-222-5p (Figure 2E), an miRNA produced from the same hairpin as the aforementioned stable miR-222-3p. Other fast turnover examples include miR-92a-1-5p and miR-125b-1-3p, whose counterparts miR-92a-3p and miR-125b-5p were stable (Supplementary Figure S3I, J and K). The fast turnover rates are not restricted to HDMYZ cells, but also in other cell types, as evident in the miRNA profiling data (Supplementary Figure S3E, F and I), and through ectopic expression of miR-222 in a different cellular background (see Supplementary Results and Supplementary Figure S5). The fast turnover behavior of miR-222-5p was further confirmed with qRT-PCR (Supplementary Figure S3G and H). We also noticed that the fast turnover miRNAs are not limited to those of low abundance. miR-21-3p, which accounts for 7.7% of total miRNA reads in this cell type, is significantly reduced after ActD treatment (Figure 2F and G).

The vast majority of fast turnover miRNAs, including miR-222-5p and miR-125b-1-3p, belong to the previously categorized miRNA* species (37). Specifically, 12 of 17 miRNAs with <25% remaining at 12 h post-ActD and 27 out of 34 miRNAs with <50% remaining can be classified as miRNA* (Figure 2D). We also noticed that some annotated non-star miRNA strands were also among the fast or ultrafast turnover miRNAs. For example, miR-4521 was nearly completely turned over within 2 h, as confirmed with qRT-PCR (Supplementary Figure S2C). However, this could possibly be due to mis-annotation of other types of small RNAs as miRNAs in the miRBase.

Taken together, our data above show that while most miRNAs are stable, a small subset of miRNAs, mostly annotated miRNA*s, has fast and ultrafast turnover rates.

### Two-step kinetics for many fast turnover-over miRNAs

**Figure 3.**
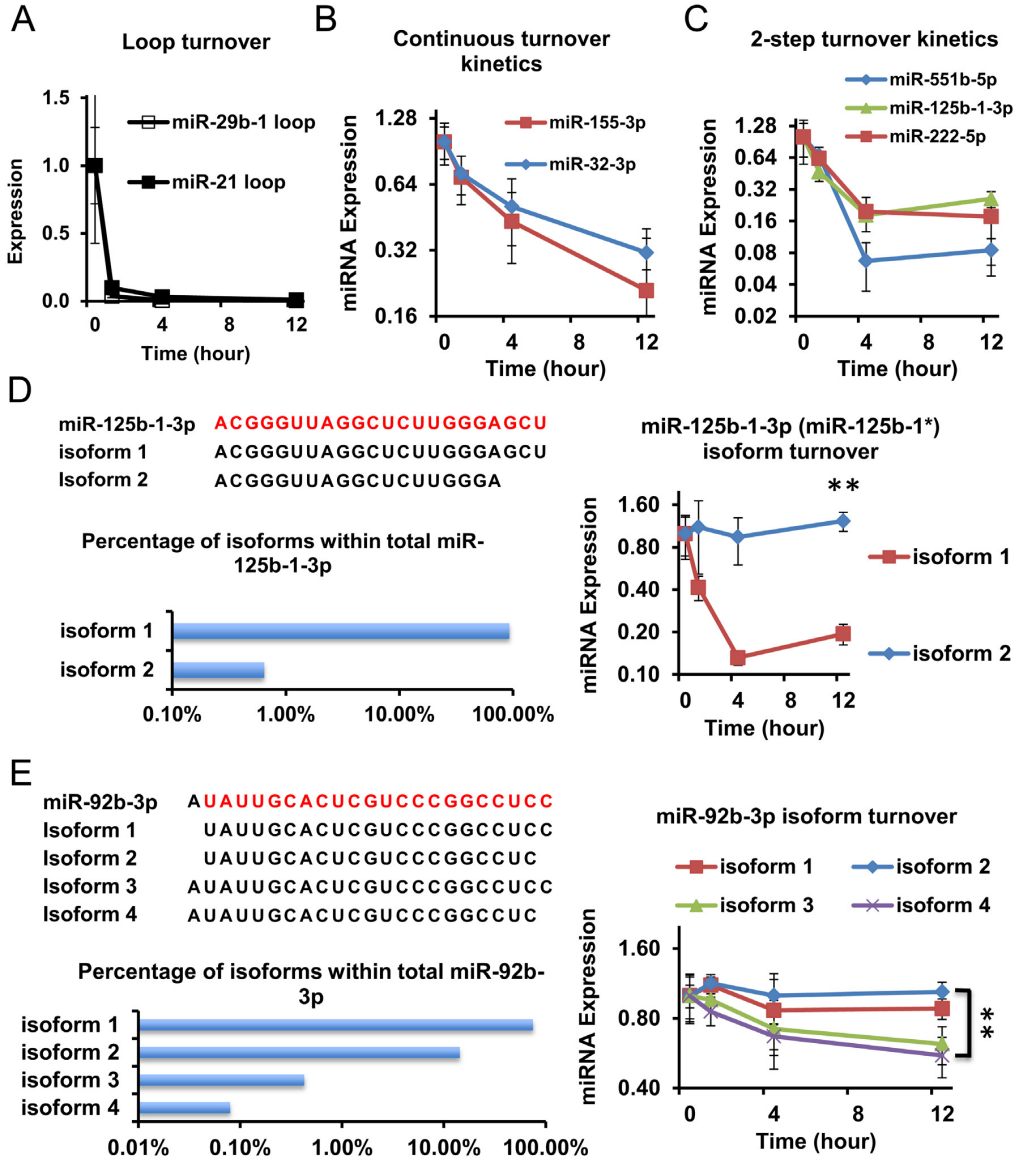
Differential turnover kinetics of miRNAs and miRNA isoforms. (**A, B, C**) Three main categories of turnover kinetics were observed in deep sequencing data. Time points post-ActD treatment are indicated. *N* = 3. Data represent miRNA expression levels relative to the 0-h level, which is set to 1. (A) Loop regions from miRNA precursors show <1-h half-lives. (B) Examples of continuous turnover kinetics: miR-155-3p and miR-32-3p are shown. (C) Examples of 2-step turnover kinetics: miR-551b-5p, miR-125b-1-3p and miR-222-5p are shown. Note the initial fast turnover phase followed by a much slower turnover kinetics. Turnover kinetics of miR-125b-1-3p (**D**) and miR-92b-3p (**E**) isoforms. Upper left panel: sequence of miRNA isoforms is shown. Mature miRNA sequences from miRBase are shown in red/lighter color, with isoform sequences shown below. Lower left panel: the relative percent abundance of indicated miRNA isoforms relative to total mapped reads to the corresponding miRNA is shown. Right panel: turnover kinetics of different miRNA isoforms after ActD treatment. *N* = 3. Error bars stand for standard deviation. **P < 0.01.

Close examination of sequencing data revealed that there are three main types of turnover kinetics observable for miRNAs with fast or ultrafast turnover rates (Supplementary Table S2). First are those that nearly completely disappeared after only 1 h of ActD treatment (half-life much less than 1 h). These miRNAs include the aforementioned annotated miR-4521 (Supplementary Figure S2C), which behave similarly to most of the loop regions produced from Dicer cleavage of miRNA precursors (Figure 3A). These results confirmed that ActD was fast-acting in our experiments, and also suggest that such annotated miRNAs may not be real miRNAs. The second class of miRNAs turned over with a relatively constant rate, with half-lives of 4 h or beyond, including miR-155-3p and miR-32-3p (Figure 3B). The turnover behaviors of such miRNAs are similar to a canonical RNA turnover phenomenon. The third class of miRNAs showed a clear pattern of two-step kinetics. Approximately fifty percent of miRNAs with fast turnover rates belong to this class (Supplementary Table S2). Examples include the aforementioned miR-21-3p (miR-21*) and miR-125b-1-3p (miR-125b-1*) (Figures 2F and 3C). These miRNAs had a fast turnover phase with half-lives close to 1 h (Figure 3C and Supplementary Figure S2D). For different miRNAs in this class, variable amounts remain after this fast turnover phase. The remaining miRNA amounts turnover with a much slower kinetics, and many of them appear stable after 4 h of ActD treatment (Figure 3C and Supplementary Figure S2D).

The data above showed that different fast turnover miRNAs had differential kinetics, with two-step kinetics observable for a significant portion of fast turnover miRNAs.

### Co-existence of multiple isoforms of the same miRNA with distinct turnover kinetics

Since multiple sequence isoforms exist for each mature miRNA (e.g. Figure 3D), we assessed turnover kinetics for miRNA isoforms. In total, there were 1036 reliably quantifiable miRNA isoforms in HDMYZ cells (see the Materials and Methods section), among which 3.5% decreased >75% and 8.5% decreased >50% in abundance after 12 h of ActD treatment (Supplementary Table S3).

We found multiple miRNAs of which different miRNA isoforms showed differential turnover kinetics. For example, we examined the sequence isoforms for the overall unstable miR-125b-1* (miR-125b-1-3p). Among the two illustrated isoforms for this miRNA, isoform 1 showed rapid turnover resulting in <20% of abundance after 12 h of ActD treatment (Figure 3D). In contrast, isoform 2 for the same miRNA, although of much lower abundance, was more stable (Figure 3D). The differential stability of the miR-125b-1* isoforms is unlikely the result of accumulation of turnover intermediates, otherwise we would expect significant increased abundance of certain isoforms (which we did not observe) given that the unstable isoform 1 represented ∼93% of sequence reads for this miRNA. Notably, the unstable miRNA isoforms also showed two-step turnover kinetics.

Interestingly, we also found that for overall stable miRNAs, there can be unstable isoforms. Examples include miR-92b-3p, a non-star and overall stable miRNA. Among the four isoforms shown in Figure 3E, while isoforms 1 and 2 were stable, isoforms 3 and 4 were less stable (Figure 3E). Together, the data above indicate the co-existence of miRNA isoforms with distinct turnover rates.

### AGO-complex-associated miRNA* fraction undergoes fast turnover

**Figure 4.**
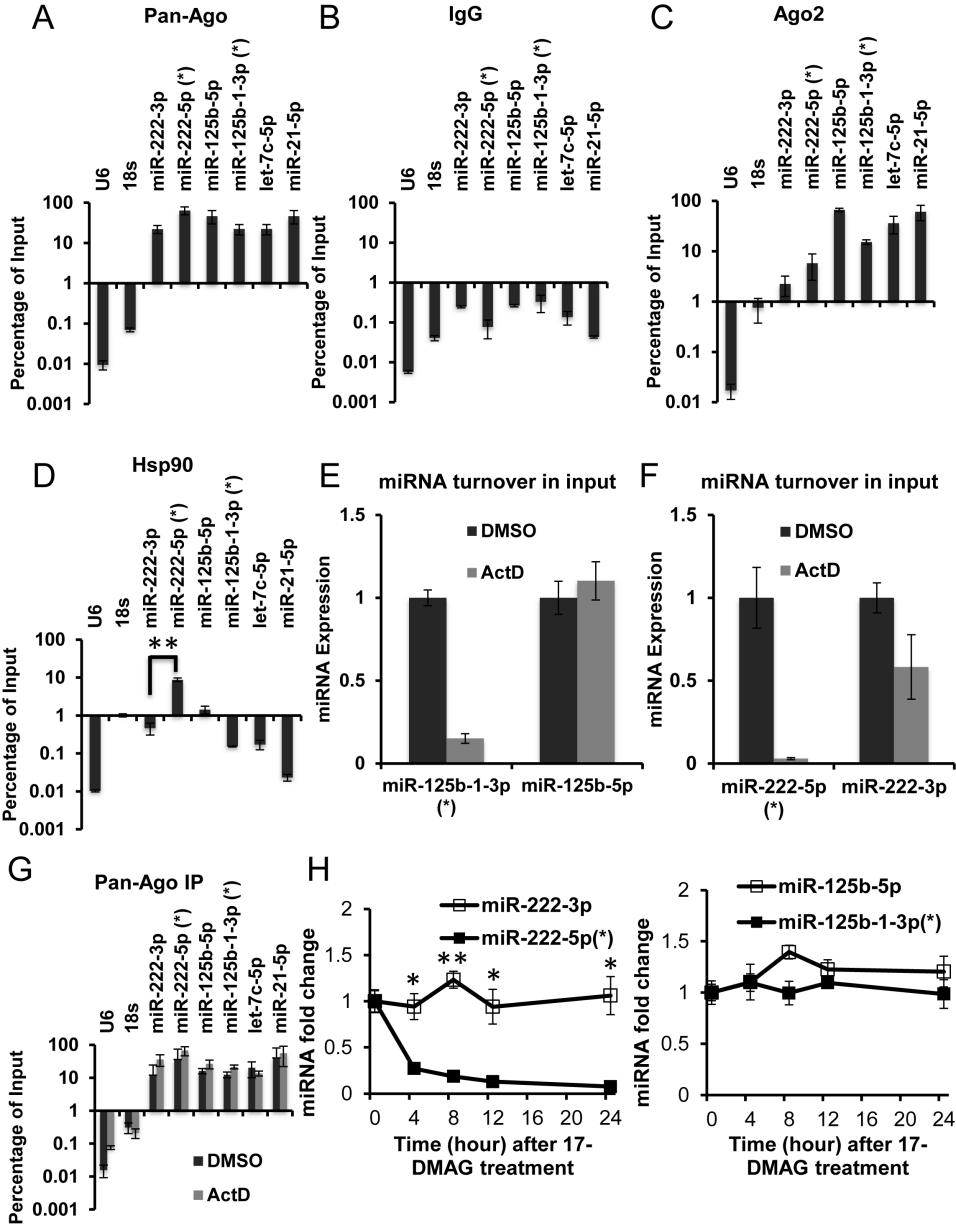
Differential HSP90 association and dependence of fast turnover miRNA*s. HDMYZ cells were immunoprecipitated (IPed) with pan-AGO (**A**), IgG (**B**), AGO2 (**C**) or HSP90 (**D**) antibodies. Levels of indicated miRNAs in the IP fraction or the input material were determined by qRT-PCR. Data represent the levels of miRNAs in the IP fraction normalized to the same fraction of input. *N* = 3. (**E–G**) HDMYZ cells were treated with vehicle control (DMSO) or ActD for 4 h. (E, F) Levels of miR-125b-1-3p (miR-125b-1*) and miR-125b-5p (E), miR-222-5p (miR-222*) and miR-222-3p (F) were determined in the input samples used for pan-AGO IP. (G) Levels of indicated miRNAs in the IP fraction were determined by qRT-PCR and normalized to the same fraction of input. *N* = 3. Levels of miR-222-5p (miR-222*) and miR-222-3p (**H**), miR-125b-1-3p (miR-125b-1*) and miR-125b-5p (**I**) were measured by qRT-PCR after 17-DMAG treatment in HDMYZ cells, at indicated time points. *N* = 3. Error bars stand for standard deviation. *P < 0.05; **P < 0.01.

To gain mechanistic insights into the fast turnover miRNAs, we used two fast turnover miRNA*s as models and asked whether such miRNAs can physically associate with AGO-containing complexes. Specifically, we examined miR-222* (miR-222-5p) and miR-125b-1* (miR-125b-1-3p). We first immunoprecipitated (IP) HDMYZ cell lysates (with triplicate) with a widely used pan-AGO antibody that recognizes all four human AGO proteins (38), and performed qRT-PCR to quantify miRNA-AGO association. The pan-AGO-IP was of good quality, because stable non-star miRNAs were pulled down with high efficiency, including miR-222-3p, miR-125b-5p, let-7c-5p and miR-21-5p (Figure 4A), whereas negative controls, such as RNU6B and 18S RNAs, were effectively depleted (Figure 4A). In addition, western analysis showed that the AGO-specific band was preferentially enriched over a non-specific band during IP with this antibody (Supplementary Figure S5F). Of surprise, both the fast-turning-over miR-222* and miR-125b-1* were enriched in the pan-AGO IP, to comparable levels as their stable non-star counterparts miR-222-3p and miR-125b-5p (Figure 4A and Supplementary Figure S5A). (Of note, variable AGO-IP efficiencies were observed for different canonical stable miRNAs, consistent with previous literature findings on AGO-IP of canonical miRNAs (39).) In contrast, none of the miRNAs were enriched in control IgG IP (Figure 4B and Supplementary Figure S5B). To avoid the potential non-specificity of pan-AGO antibody complicating data interpretation, we also performed similar experiments with another antibody specific to AGO2 (40) and reached a similar conclusion (Figure 4C and Supplementary Figure S5C and F). These results indicate that the unstable miRNA*s are associated with AGO complex.

We next asked whether the AGO-associated fraction of miR-222-5p or miR-125b-1-3p underwent rapid turnover. We treated HDMYZ cells for 4 h with ActD or vehicle control (DMSO), before cell lysis and immunoprecipitation. As expected, ActD treatment strongly decreased the intracellular miR-222* and miR-125b-1* levels to 3% and 15% of the original, respectively (Figure 4E and F). Despite this drop of miRNAs in input, the AGO-IP fraction relative to input was similar to vehicle-treated cells for these fast turnover miRNAs, or other stable miRNAs such as miR-222-3p, miR-125b-5p, let-7c-5p and miR-21-5p (Figure 4G and Supplementary Figure S5E). These data mean that similar percentages of the unstable miR-222* and miR-125b-1* were associated with AGO, even though total unstable miRNA levels dropped sharply after ActD treatment, indicating that the AGO-associated forms of miRNA*s underwent rapid turnover. Such turnover may occur while the miRNA*s were associated with AGO complex, or may occur after their dissociation from AGO complex, two possibilities that will be interesting to determine in future studies.

### Differential requirement of HSP90 for fast-turnover miRNA*s

Our results above indicate that AGO-association status by itself cannot explain the fast or slow turnover phenomena. One possibility is that under steady state in HDMYZ cells, the unstable miR-222* and miR-125b-1* mostly exist as an intermediate during the loading process into AGO complex. Recent studies indicate the essential role of HSP90 in the miRNA maturation process for loading miRNA duplex onto AGO (6–11). We thus immunoprecipitated HDMYZ cell lysate with an HSP90 antibody. We could not detect obvious enrichment of the stable miR-222-3p, miR-125b-5p, let-7c-5p or miR-21-5p in the HSP90-IP fraction (Figure 4D and Supplementary Figure S5D). This is expected, since most of such stable miRNAs are likely loaded into AGO, and only a small fraction of them may be during active loading process. Consistent with the unstable miR-222* existing as an intermediate during the AGO-loading process, we observed that miR-222-5p (miR-222*) was enriched in the HSP90-IP fraction (Figure 4D and Supplementary Figure S5D). Surprisingly, another unstable miRNA, miR-125b-1*, was not enriched (Figure 4D and Supplementary Figure S5D). These indicate differential binding of fast turnover miRNAs to HSP90-containing complexes.

Prompted by the differential binding, we asked whether there are differential functional requirement of HSP90 for the two unstable miRNA*s. We reasoned that since both miR-222* and miR-125b-1* are associated with AGO complex, the current model of miRNA biogenesis dictates that HSP90 would be required before or at this AGO-association stage, both leading to decreased unstable miRNA abundance when HSP90 is inhibited. For stable non-star miRNAs, we do not expect major changes in miRNA abundance with HSP90 inhibition (10), because the fraction of miRNAs undergoing active loading within the stable miRNA pool is likely small. We treated HDMYZ cells with 17-DMAG, a specific HSP90 inhibitor (41). The miR-222* level decreased more than 75% at 4 h after HSP90 inhibition and near completely at later time points (Figure 4H), whereas the stable counterpart miR-222-3p was largely unchanged. For miR-125b-1-derived miRNAs, however, both the stable miR-125b-5p and the unstable miR-125b-1* showed much less decrease, if any (Figure 4I), consistent with our inability to detect miR-125b-1-3p in association with HSP90-containing complex.

To further confirm the differential sensitivity of miR-222* and miR-125b-1* toward HSP90 inhibition, we treated HDMYZ cells with a series of concentrations of 17-DMAG or 17-AAG, another HSP90-specific inhibitor (42) for 4 h. We observed substantial difference between miR-222* and miR-125b-1* (Supplementary Figure S5G and H, ∼100-fold concentration difference). While miR-222* could be downregulated by HSP90 inhibitors at low concentrations, miR-125b-1* was mainly downregulated by HSP90 inhibitors at high concentrations that are associated with morphological cellular toxicity.

Our data above demonstrate endogenous miR-222* in association with HSP90, suggesting the existence of an HSP90/AGO intermediate for AGO loading of miR-222 *in vivo*. Our data also demonstrate that while miR-222-5p showed both HSP90 association and HSP90 dependence, miR-125b-1-3p showed AGO association but neither HSP90 association nor dependence, indicating differential requirement of HSP90.

### Enhanced miR-222-5p loading increases its stability

According to the prevailing model (6–11), HSP90 prepares AGO for the miRNA duplex loading process, but is not required afterward. The association of miR-222* with AGO and HSP90 suggests that such AGO association represents a state of loading intermediate. If this is true, we would predict that enhancing miR-222* AGO loading could improve its stability.

**Figure 5.**
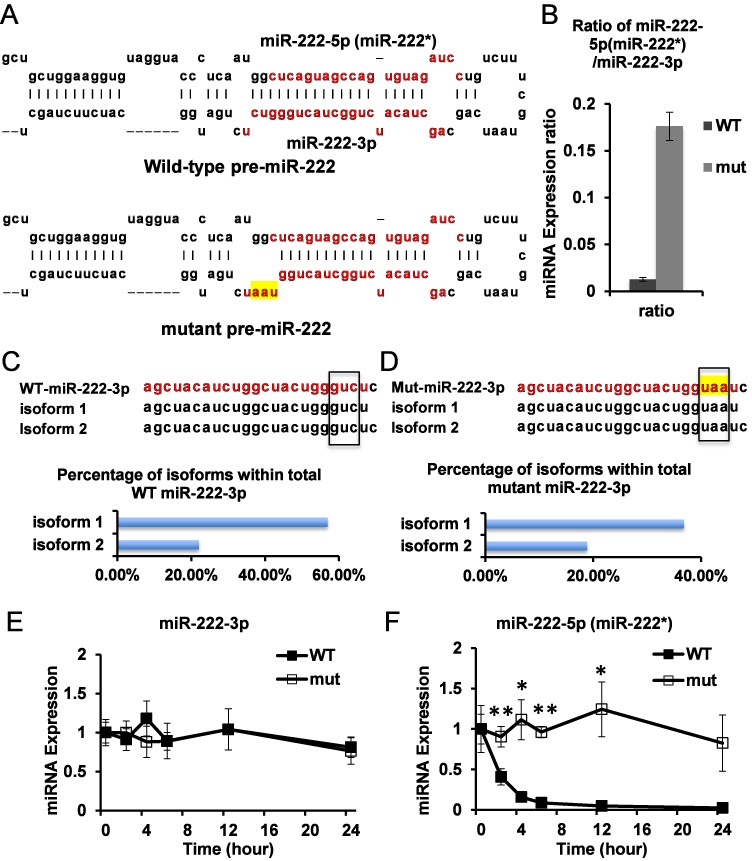
Enhanced miR-222-5p loading increases its stability. (**A**) Schematic of miR-222 precursor and mutation. Note that mutation only affects miR-222-3p sequence but not miR-222-5p (miR-222*) sequence. Mutated nucleotides are highlighted. Red/lighter colored letters represent the most abundant isoform of corresponding mature miRNA based on deep sequencing data. (**B**) Ratio of miR-222-5p versus miR-222-3p expression level in MCF7 cells transduced with wild-type (WT) and mutant (mut) miR-222 precursor. Expression levels of miR-222-3p and miR-222-5p (miR-222*) were determined by qRT-PCR. Note that for miR-222-3p, the qPCR primer recognizes both WT and mut forms. The most abundant miRNA isoforms are shown, corresponding to wild-type miR-222-3p (**C**) and mutant miR-222-3p (**D**). Upper panel: sequences of miRNA isoforms are shown. Sequences of the most abundant miRNA isoform are shown in red/lighter color, with detected isoform sequences shown below. Mutated nucleotides are highlighted. The positions of the mutation are indicated with the rectangles. Lower panel: the percent abundance of the top two isoforms relative to total mapped reads to the corresponding miRNA is shown. WT and mut miR-222 expressing MCF-7 cells were treated with ActD for the indicated time. The levels of WT and mut miR-222-3p (**E**) and miR-222-5p (miR-222*) (**F**) were determined by qRT-PCR. *N* = 3. Error bars stand for standard deviation. *P < 0.05; **P < 0.01.

Since the loading efficiency of miR-222* is regulated by the thermostability of the two ends of miRNA/miRNA* duplex (14), we specifically mutated three nucleotides in the miR-222 hairpin precursor, at the 3′ end of the stable miR-222-3p, so that the weakened thermostability at this end would shift to loading more miR-222* (Figure 5A). Importantly, this mutation design does not impact the 5′ sequence of the miR-222 hairpin, from which unstable miR-222* is produced. The wild-type and mutant miR-222 genes were transduced into MCF7 cells, which had low endogenous levels of miR-222 miRNAs to allow us to determine the behavior of ectopically expressed miR-222 miRNAs. Sequencing results support that mutant miR-222 was similarly processed as wild-type miR-222, without major alterations in Drosha or Dicer cleavage sites (Figure 5C and D). We then measured the half-lives of miR-222* and miR-222-3p in the presence of ActD. Of note, we used a primer that recognizes both wild-type and mutant miR-222-3p to measure its abundance. As expected, the mutation resulted in elevated miR-222* and decreased miR-222-3p levels, leading to a 14-fold increase of the relative abundance of miR-222* to miR-222-3p in the cells, supporting that we achieved enhanced loading of miR-222* (Figure 5B and Supplementary Figure S2E and F). While miR-222* from the wild-type construct showed ultrafast turnover kinetics, miR-222* from the mutant construct was significantly stabilized, without a major impact on the stability of miR-222-3p (Figure 5E and F). The above results indicate that without any sequence mutation in miR-222*, the turnover rate of miR-222* can be altered with enhanced RISC loading. These data support the model that AGO-complex-associated unstable miR-222* in wild-type cells represents a loading intermediate.

### Fast turnover miRNAs are enriched for multiple sequence features

**Figure 6.**
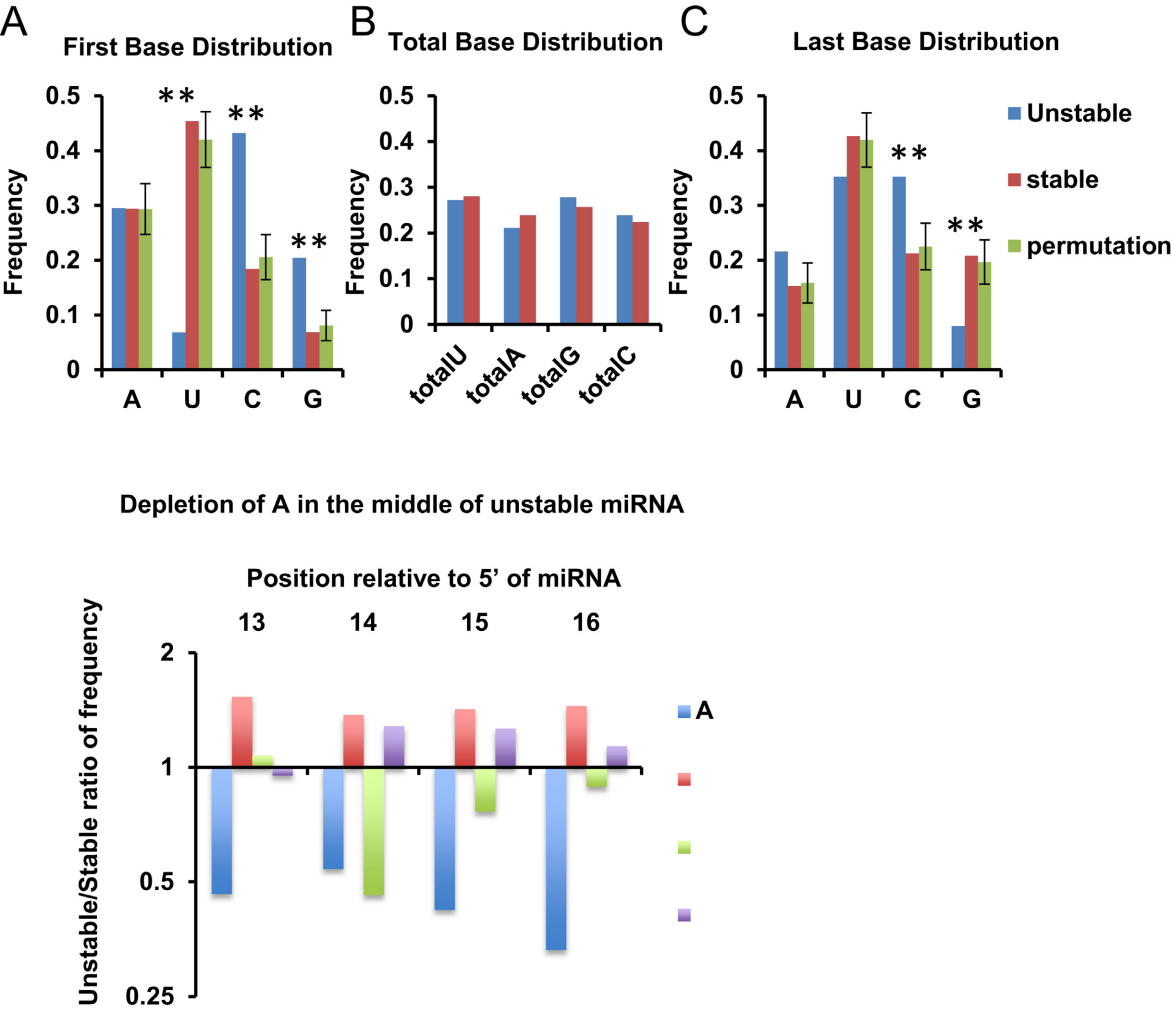
Sequence features of fast turnover miRNA isoforms. Nucleotide distribution of fast turnover (<50% remaining after 12 h of ActD treatment; see the Materials and Methods section) and slow turnover (≥75% remaining after 12 h of ActD treatment) miRNA isoforms was compared. Data collected from 1036 reliably quantifiable miRNA isoform sequences in HDMYZ deep sequencing results. First-base distribution (**A**), total base-pair distribution (**B**), last-base distribution (**C**) and an A-rich motif from bases 13 to 16 of miRNAs (**D**) are shown. Data were represented either as frequency or frequency ratio between unstable and stable miRNA isoforms. For (A) and (C), permutation analyses were performed with error bars representing standard deviation from 10 000 permutations. *P < 0.05; **P < 0.01.

To assess whether the AGO loading restraints for miR-222* stability are generalizable, we examined next-generation sequencing data and asked whether fast-turning-over miRNA isoforms are enriched for sequence features unfriendly for AGO loading. Since the first-base of U or A was believed to be more efficient for human AGO2 binding (17), we compared miRNA isoforms that showed relatively fast turnover (with <50% abundance or <25% after 12 h of ActD) with those that were more stable. The fast turnover isoforms were significantly enriched for first-base G and C, but were depleted for the occurrence of first-base U (Figure 6A and Supplementary Figure S4A). In contrast, the total base composition was similar between miRNA isoforms with different turnover kinetics (Figure 6B). This result is consistent with AGO loading playing a role in determining miRNA stability.

However, first-base A, which also favors AGO2 binding (17), was present at similar levels in both stable and unstable fractions (Figure 6A and Supplementary Figure S4A). In addition, there are unstable miRNAs with first-base U and stable miRNAs with first-base of G or C. These suggest that additional features other than first-base composition can be associated with fast turnover miRNAs. We expanded the analysis to other bases in the sequences of miRNA isoforms and found two additional features associated with fast turnover miRNAs. First, we identified that fast turnover miRNAs had a skewed last base distribution, with significant depletion of G and enrichment of C (Figure 6C and Supplementary Figure S4B). Of note, we restricted analyses to sequence reads that mapped perfectly to miRNA hairpins, so that untemplated 3′ nucleotide addition (43,44) is unlikely a major contributor to this difference. Second, we found that an A-rich motif from bases 13 to 16 of the miRNA sequence is significantly depleted in unstable miRNA sequences (Figure 6D and Supplementary Figure S4C). We also examined isoform length distribution (Supplementary Figure S4D and E). Although fast turnover miRNA isoforms are slightly shorter than stable ones, both stable and unstable isoforms showed a major peak of 22 nt in length, suggesting sequence length is not a major contributor to turnover kinetics (Supplementary Figure S4D and E).

Together the data above support a general role of efficient AGO loading in determining the turnover kinetics, but also reveal additional sequence features for fast turnover miRNAs.

## DISCUSSION

Our study revealed the miRNA turnover landscape in cultured mammalian cells under homeostatic conditions without cell cycle synchronization. Consistent with current literature (18–20), we found that most miRNAs are stable. Among the less stable miRNAs, the vast majority belongs to the miRNA* category. This finding corroborates and extends previous knowledge obtained on specific mammalian or *Caenorhabditis elegans* miRNA*s (19,23,45). However, unlike previous reports, we comprehensively characterized the turnover kinetics of mammalian miRNA*s, many of which were much faster than those reported in worm (23). This difference may be due to technical differences in the administration of the inhibitors (23). This difference may also be due to differences in the miRNA machineries in different species. For example, a study on rules for miRNA target recognition suggests that the miRNA machinery in *C. elegans* may be more tolerant of G•U base pairing and mismatches in the seed region (46).

Our study identified several sequence features enriched for fast turnover miRNAs. While miRNA targets and several exonucleases and proteins were found to regulate miRNA turnover in multiple organisms (19), the sequence determinants in miRNA themselves have not been well understood. Our sequence feature analysis was carried out on the level of miRNA isoforms, because we showed that fast turnover miRNAs can have stable isoforms, and stable miRNAs can have fast turnover isoforms. We found that the first-base of fast turnover miRNA isoforms is enriched for G and C, but depleted for U. This is consistent with AGO2 binding preference for the 5′-base (17). However, as we also indicated, this first-base bias cannot fully explain the fast turnover behavior of all miRNA sequences. In addition to the 5′ sequence features, we also found that certain 3′ base compositions and a central A-rich motif are associated with fast turnover miRNA isoforms. Our unpublished results from another cell line, K562, reveal the same sequence features for fast turnover miRNAs, supporting that these sequence features are not HDMYZ-cell-dependent. Whether or not such sequence features play a functional role in determining miRNA stability or loading into AGO is an interesting question that should be examined with careful details in the future. The sequence features that we identified are different from previous findings on 3′ non-templated additions, which demonstrate that untemplated U addition destabilizes miRNAs and non-templated A makes miRNA more stable in a specific case (43,44,47). In addition, specific sequence motifs in the 3′ or middle of miRNAs have been reported to regulate turnover of specific miRNAs (48–50). In our study, the 3′ last-base feature we identified is not on non-templated nucleotide, but for sequences that perfectly match genomic sequence. We also attempted to examine the association of turnover with 3′ non-templated nucleotides, but due to the small number of sequence isoforms with 3′ non-templated addition, we were not able to derive statistically significant results. Furthermore, the central A-rich motif reported here has not been reported before. Together with the 5′ first-base feature observed in this study, we speculate that avoiding the sequence features for fast turnover miRNAs may help design more potent siRNAs for experimental or therapeutic purposes.

**Figure 7.**
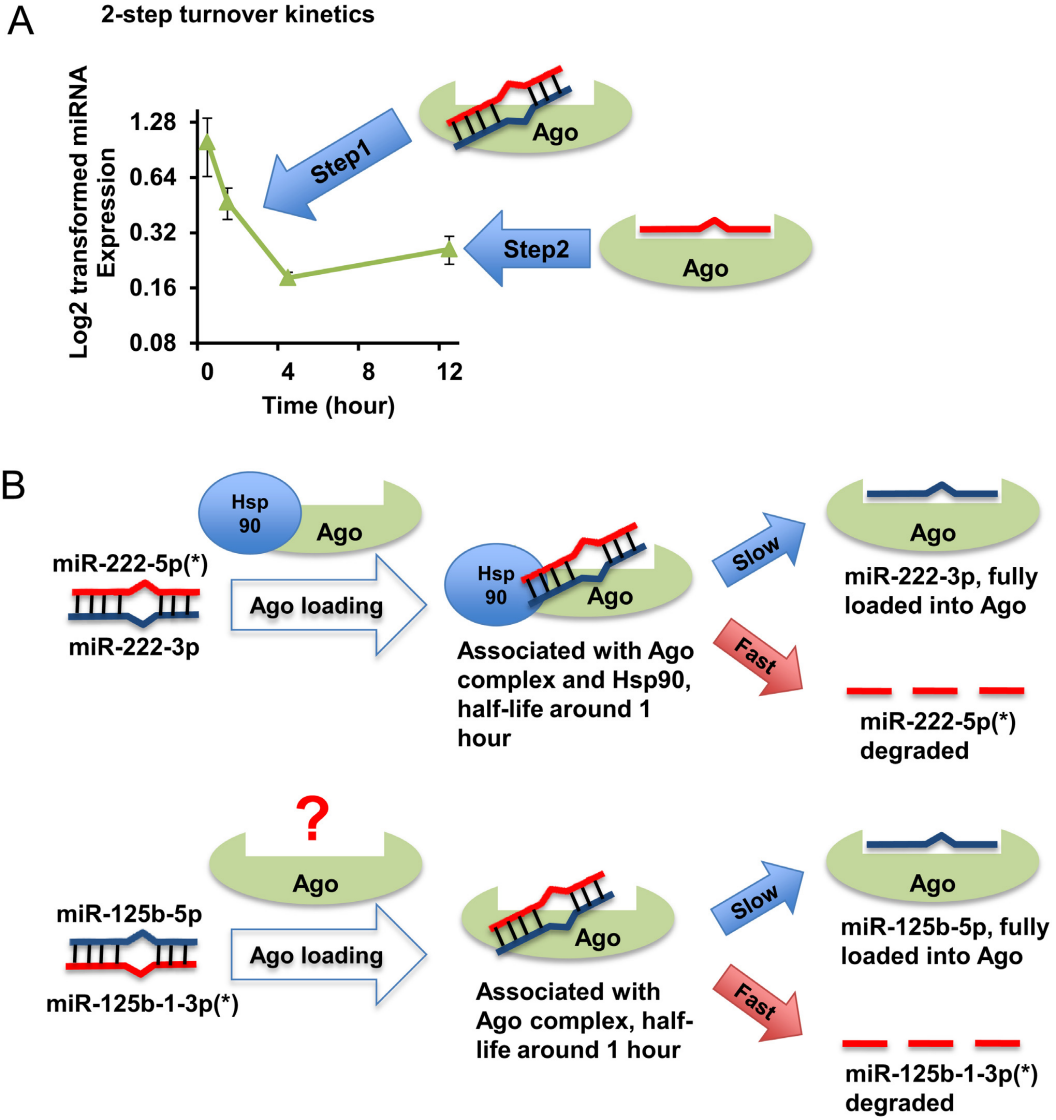
Models of miRNA turnover and differential HSP90 requirement. (**A**) Model of the two-step turnover kinetics. The AGO-associated but not fully loaded miRNA* pool has a fast turnover rate, resulting in the fast turnover phase; a minor fraction of fully AGO-loaded miRNA* results in the slow turnover phase. (**B**) Suggested models of miR-222-5p (miR-222*) and miR-125b-1-3p (miR-125b-1*) turnover. For miR-222*, most of the molecules in the cell are associated with AGO–HSP90 complex as intermediate during miRNA duplex loading; the level of miR-222* is sensitive to HSP90 inhibition. For miR-125b-1*, most of the molecules in the cell are associated with AGO but not HSP90 (may involve other chaperons or unknown factors, reflected by a question mark); the level of mir-125b-1* is not sensitive to HSP90 inhibition.

In our study, we also found that the turnover of many miRNA*s displays a two-phase kinetics, with the fast phase showing ∼1 h of half-life followed by a much more stable phase. This kinetics can be explained by a model (Figure 7A), as the fast turnover phase reveals rapid turnover of miRNAs associated with but not fully loaded into AGO, whereas the slower phase potentially reflects the turnover of fully AGO-loaded miRNAs. Our data with miR-222 mutation further support that the majority of miR-222* is only associated with AGO, but not fully loaded. Thus, our data revealed that the *in vivo* half-life of this duplex association step with AGO is about 1 h for miR-222*, and also caution the use of AGO-association status to define functional miRNAs. Given that many other miRNA*s showed similar first step turnover kinetics, this conclusion might be generalizable to other miRNA*s in HDMYZ cells. Our data also revealed that the loop regions miRNA precursors had a much shorter half-life than 1 h. These data suggest that although miR-222* has a short half-life, its association with AGO protects it from degradation mechanism(s) that targets unprotected single-stranded small RNAs. In addition, this two-step kinetics can also help explain previously identified fast turnover miRNAs with poorly understood mechanisms. For example, it was shown that several miRNAs demonstrate signal-dependent ultrafast turnover in retina, many of which display a two-step kinetics (20). It is thus possible that signal-dependent dislodging of miRNAs from AGO-containing complexes, or a signal-dependent unknown mechanism that protects AGO-associated but not loaded miRNAs, may explain this turnover behavior.

Our study provides evidence for HSP90 association of endogenous miRNAs and also unexpectedly reveals differential HSP90-depdence for the levels of different miRNA*s, suggesting that there might be a sorting mechanism for different miRNA duplexes into different cellular AGO or AGO/chaperon pools. The behavior of miR-222* provides additional support for the prevailing model in which HSP90 is required for the loading of miRNA duplex onto AGO. Specifically, miR-222* co-immunoprecipitated with both AGO and HSP90, and treatment of HSP90 inhibitor quickly and nearly completely reduced miR-222* abundance in cells. These data suggest that the vast majority, if not all, of miR-222 miRNAs went through an HSP90 intermediate for loading. While this model is consistent with previously published *in vitro* biochemical data demonstrating the role of HSP90 in the miRNA duplex loading process (6–11), our data here alone are not definitive proof that miR-222 duplex loading is through an HSP90 intermediate. It is conceivable that future experiments utilizing recombinant biochemical assays (such as those in (7)) can provide important *in vitro* evidence further supporting HSP90-dependent loading of miR-222 duplexes. It can be much more challenging, however, to gather direct *in vivo* evidence for HSP90-mediated AGO loading. Due to the many clients of HSP90 *in vivo*, it is difficult to rule out contributions from secondary effects of HSP90 inhibition.

In contrast to miR-222*, although miR-125b-1* can also co-immunoprecipitate with AGO, we did not detect its association with HSP90. If HSP90 is required for the loading of all miR-125b-1 duplex molecules onto AGO, we could then predict that the miR-125b-1*-AGO complex exists in a post-HSP90-binding step, which would, in turn, predict that the HSP90 inhibitor will also rapidly reduce miR-125b-1* abundance, because most of the miR-125b-1* is fast turnover. However, we showed that miR-125b-1* abundance is much less reduced, if any, in comparison with miR-222*, when treated with HSP90 inhibitor. Whether additional miRNAs behave like miR-125b-1 will be an interesting question for further investigation. It is possible that other molecular chaperons facilitate the loading of miR-125b-1, which may partially explain the decrease of the miR-125b-1* level after the treatment with high concentrations of HSP90 inhibitor (the HSP90 inhibitor may target other cellular chaperons at this high concentration). Besides HSP90, biochemical pull-down experiments with ectopically expressed AGO proteins revealed several additional members of the molecular chaperon family (7,51,52). However, the lack of good IP antibodies and/or specific inhibitors, and the fact that some of such chaperons are known to be quite ‘sticky’, precluded us from examining such candidates in detail in this study. Future experiments utilizing recombinant biochemical assays will help to define whether the loading miR-125b-1 duplex constitutes a separate pathway, and which protein components may be required.

## SUPPLEMENTARY DATA

Supplementary Data are available at NAR Online.

SUPPLEMENTARY DATA
