# Supplementary material for: Characterization of the mammalian miRNA turnover landscape
Source: Nucleic Acids Res. 2015 Feb 4;43(4):2326–41. doi: 10.1093/nar/gkv057 (PMC4344502; doi:10.1093/nar/gkv057)
Supplement: SUPPLEMENTARY DATA [file supp_43_4_2326__index.html]

Characterization of the mammalian miRNA turnover landscape — Characterization of the mammalian miRNA turnover landscape — SUPPLEMENTARY DATA 

# Characterization of the mammalian miRNA turnover landscape

## SUPPLEMENTARY DATA

**Files in this Data Supplement:**

- SUPPLEMENTARY DATA
- SUPPLEMENTARY DATA
- SUPPLEMENTARY DATA
- SUPPLEMENTARY DATA
- SUPPLEMENTARY DATA
