# Supplementary material for: Characterization of the mammalian miRNA turnover landscape
Source: Nucleic Acids Res. 2015 Feb 4;43(4):2326–41. doi: 10.1093/nar/gkv057 (PMC4344502; doi:10.1093/nar/gkv057)
Supplement: SUPPLEMENTARY DATA [file supp_gkv057_nar-02456-y-2014-File003.pdf]

Supplementary Materials

Characterization of the Mammalian miRNA Turnover Landscape

Yanwen Guo, Jun Liu, Sarah J. Elfenbein, Yinghong Ma, Mei Zhong, Caihong Qiu, Ye Ding and Jun Lu

This file includes:

Supplementary Results
Supplementary Materials and Methods
Supplementary Figures and Legends
Supplementary References

Supplementary Results

The fast turnover rates are an intrinsic property of relevant miRNAs

For fast-turnover miRNAs, we asked whether the fast turnover rate is an intrinsic property of the relevant miRNAs. This is because global transcription inhibition, in theory, may affect potential miRNA "protectors" if they themselves turnover fast.

We modeled miR-222-5p (miR-222*) and miR-23a-5p (miR-23a*), and used a tet-off system to ectopically express them in cell lines that normally do not express the relevant miRNA at high levels. With the inducible expression, we could shut down the ectopic miRNA expression and follow their turnover without affecting global transcription in the cells (Fig S6A). Specifically, we transduced MCF-7 cells with tTA and a miR-222-hairpin-expressing vector under the control of tetO promoter. After establishing ectopic expression of miR-222-5p and miR-222-3p, we added Doxycycline to shut down the ectopic transcription. Because this Tet-off system may not stop transcription as instantaneously as chemical transcriptional inhibitors, we first evaluated its performance using a short-lived luciferase, known to have very short RNA and protein half-lives (1) (Fig S6D). The level of luciferase RNA dropped ~50% after 4 hours and ~75% after 6 hours. In comparison, the level of the fast-turning-over miR-222-5p decreased to background level after 6 hours (Fig S6B), even faster than luciferase. In contrast, the stable miR-222-3p only showed ~20% drop after 24 hours (Fig S6B), a level expected because the proliferation of MCF-7 cells effectively diluted the pre-made stable miR-222-3p. Similar results were obtained with miR-23a in Raji cells (Fig S6C), a cell line with faster proliferation. These data indicate that the fast-turnover rates of these miRNAs are intrinsic properties of the miRNAs themselves.

In addition, we found it highly unlikely that active extracellular secretion underlies rapid miR-222-5p turnover. In established HDMYZ culture, the amount of miR-222-5p in the cell medium was only < 0.05% of the amount in cells (Fig S6E, S6F, S6G). In

addition, the level of miR-222-5p in medium did not significantly change after cells were treated with ActD, whereas the intracellular level of miR-222-5p drastically dropped after treatment (Fig S6E). Together, these data support the notion that the fast-turnover rates are properties of these miRNAs and are mediated through an intracellular mechanism.

Supplementary Materials and Methods

Constructs

pMSCV-tTA construct was cloned by PCR-amplifying tTA from pREV-Tet-off plasmid (Clontech), then inserted into pDONR-221 through GateWay cloning (Invitrogen). tTA fragment was further cloned into pMSCV-blast vector through LR recombination reaction (Invitrogen). pMSCV-blast was created by replacing GFP in pMIRWAY-GFP vector (2) with blasticidin resistance gene.

The inducible lentiviral miR-222 and miR-23a plasmid was cloned similarly as published (2), except that puromycin resistance gene replaced GFP. miR-222 was amplified from human genomic DNA with primers gtcactcagtcagtatctgttg and ttcataaaaccttgaaggttccc first, then amplified and gel purified again with primers ggggacaagtttgtacaaaaaagcaggctacgtacatgggaatattgtc and ggggaccactttgtacaagaaagctgg-gtcccagctgataatgttggac, and then GateWay cloning into the lenti-inducible vector pFU-tetO-PGK-puro. Similarly, miR-23a was amplified with primers tgcaagttgctgtagcctcctt and actgtgaacacgacttggtgtg first, then amplified with primers ggggacaagtttgtacaaaaaagcaggct-gaacggagggcacagcta and ggggaccactttgtacaagaaag-ctgggtccaggcacaggcttcggg, and inserted into pFU-tetO-PGK-puro. Luciferase reporter gene (luc2P) was amplified with ggggacaactttgtacaaaaaagttggcaccatggaaga-tgccaaaaacattaag and

ggggacaactttgtacaagaaagttggcaaggccttagacgttgatcctg from pGL4 construct (Promega), and inserted into pFU-tetO-PGK-puro.

Mutant miR-222 expression construct was cloned by PCR-based site directed mutagenesis based on the miR-222 expression construct. The following primer was used with mutated bases underlined: ctggctactggtaatctgatggcat. Wild-type and mutant miR-222 gene was cloned into pMIRWAY-GFP construct described in (3).

Quantitative RT-PCR

For c-myc and 18S rRNA, total RNA went through reverse transcription reaction with Invitrogen Superscript III cDNA synthesis kit following manufacturer's protocol. qPCR was performed with Taqman (ABI) gene expression assays (c-myc: Hs00153408_m1, 18s: 4319413E) using Biorad C1000 Thermal Cycler. For 18s measurement in IP samples, Taqman qPCR was performed on Qiagen miScript miRNA reverse transcription product (with HiFlex buffer). For miRNA qPCR, both Taqman miRNA expression assays (for miR-222-3p: 002276, miR-222-5p: 002097, miR-23a-3p: 000399, miR-23a-5p: 002439) and Qiagen miScript miRNA PCR assays (for miR-222-3p: MS00007609, miR-222-5p: MS00009177, miR-125b: MS00006629, miR-125b-3p: MS00008561, miR-4521, RNU6B (U6): MS00033740) were used, following manufacturers' protocols. For let-7c-5p, miR-21-5p and miR-4521 we used the following primer for qPCR and the reverse transcription products from Qiagen miScript reactions: tgaggtagtaggttgtatggtt (let-7c-5p), tagcttatcagactgatgttga (miR-21-5p), gctaaggaagtcctgtgctcag (miR-4521). We used a primer (agctacatctggctactgg) to measure both wild-type and mutant miR-222-3p with Qiagen miScript miRNA PCR assays.

To normalize qRT-PCR data, miRNA or c-myc level was first normalized to 18s or U6 level from the same sample, then normalized to their respective 0 hour samples or control.

For IP samples, the data for miRNAs, 18s and U6 level were normalized based on sample fraction, so that the data reflect their levels relative to those in input (i.e. 100% means complete recovery of the relevant RNA species from the IP experiment).

Measurement of miRNA turnover with an inducible system

MCF-7 cells and Raji cells were transduced with pMSCV-tTA-PGK-blast virus and selected for transduced cells with 15 ug/mL Blastocidin. tTA-expressing MCF-7 and Raji cells were then transduced with pFU-tetO-PGK- puro-miR-222 and pFU-tetO-PGK-puro-miR-23a, respectively, and selected with 2ug/ml puromycin. tTA-expressing MCF-7 cells were also transduced with pFU-tetO-PGK-puro-luc2P to show efficiency of transcription inhibition. Cells were maintained in culture medium without doxycycline. To inhibit exogenous miRNA expression, 4ug/ml Doxycycline was added for 6 and 24 hours.

Measurement of mutant miR-222 turnover

MCF-7 cells were transduced with retroviruses for pMIRWAY-GFP-WT-miR-222 or pMIRWAY-GFP-mut-miR-222. To control for similar transduction levels for wild-type and mutant miR-222 viruses, we controlled the transduction rate to be ~50% for both viruses. Transduced cell pools were then sorted by FACS, gating on GFP+ cells with similar GFP levels between wild-type and mutant miR-222. Cells were then treated with 10ug/ml ActD for 0, 2, 4, 6, 12, 24 hours.

Measurement of miR-222 secretion

HDMYZ cells were treated with 2.4ug/ml ActD or the same volume of DMSO for 8 hours. The same fraction of HDMYZ cells and cell culture supernatant were collected in TRIzol and TRIzol LS reagent for RNA extraction (e.g. 20% of cells and 20% of medium). All the RNA samples were diluted in 7ul of water and 5.8ul of RNA from each sample was used in Qiagen miScript RT-qPCR system. The miR-222-3p, miR-222-5p and U6 level in the cells were normalized to their level in cells with DMSO treatment.

Supplementary Figures and Legends

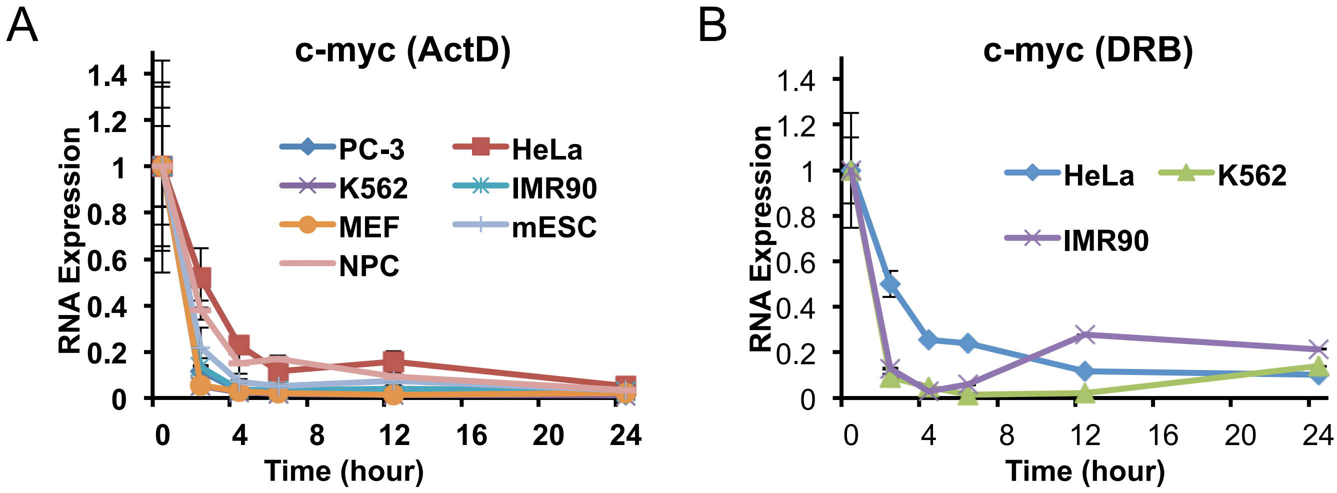

Guo et al. Figure S1

Figure S1. Effective downregulation of c-myc by transcription inhibitors. Levels of c-myc RNA post ActD **(A)** and DRB **(B)** treatment in the indicated cell types for the indicated time points were measured by qRT-PCR (Taqman). Data were normalized to 0 hour time point. N=2 for ActD treated samples. N=3 for DRB treated samples. Error bars stand for standard deviations with N=3, or average deviations from median with N=2.

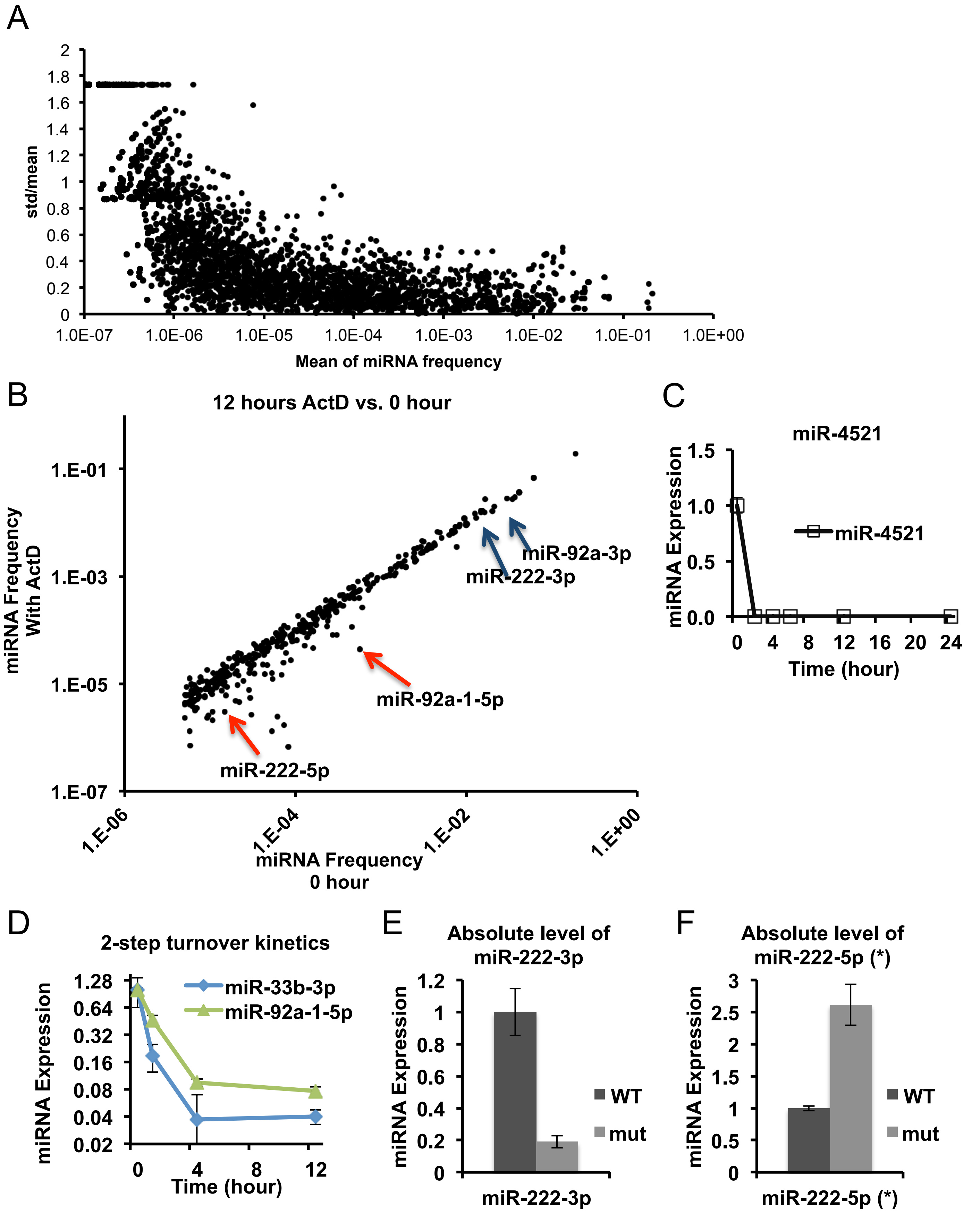

Guo et al. Figure S2

Figure S2. Deep sequencing reveals different miRNA turnover kinetics. (A) Standard deviation divided by mean of miRNA frequency vs. miRNA expression level was plotted to show the variation of deep sequencing data. Each dot represents a single annotated mature miRNA for a specific time point. Data from all time points are shown. **(B)** Dot plots of normalized frequency of miRNA sequencing reads are shown, for comparison between HDMYZ cells without treatment and those treated with ActD for 12 hours. Data represent the mean from biological triplicates. Each dot represents one mature annotated miRNA. Two fast turnover miRNAs (miR-222-5p and miR-92a-1-5p) are indicated with red arrows, their slow turnover counterparts (miR-222-3p and miR-92a-3p) are indicated with blue arrows. N=3. **(C)** Levels of miR-4521 were measured by qRT-PCR after ActD treatment in HDMYZ cells. N=2. **(D)** Turnover kinetics of miR-33b-3p and miR-92a-1-5p are additional examples of 2-step turnover kinetics. N=3. **(E, F)** Levels of miR-222-3p (E) and miR-222-5p (*) (F) measured by qRT-PCR normalized with GFP level. N=3. Data represent miRNA frequency by deep sequencing. N=3. Error bars stand for standard deviations with N=3, or average deviations from median with N=2.

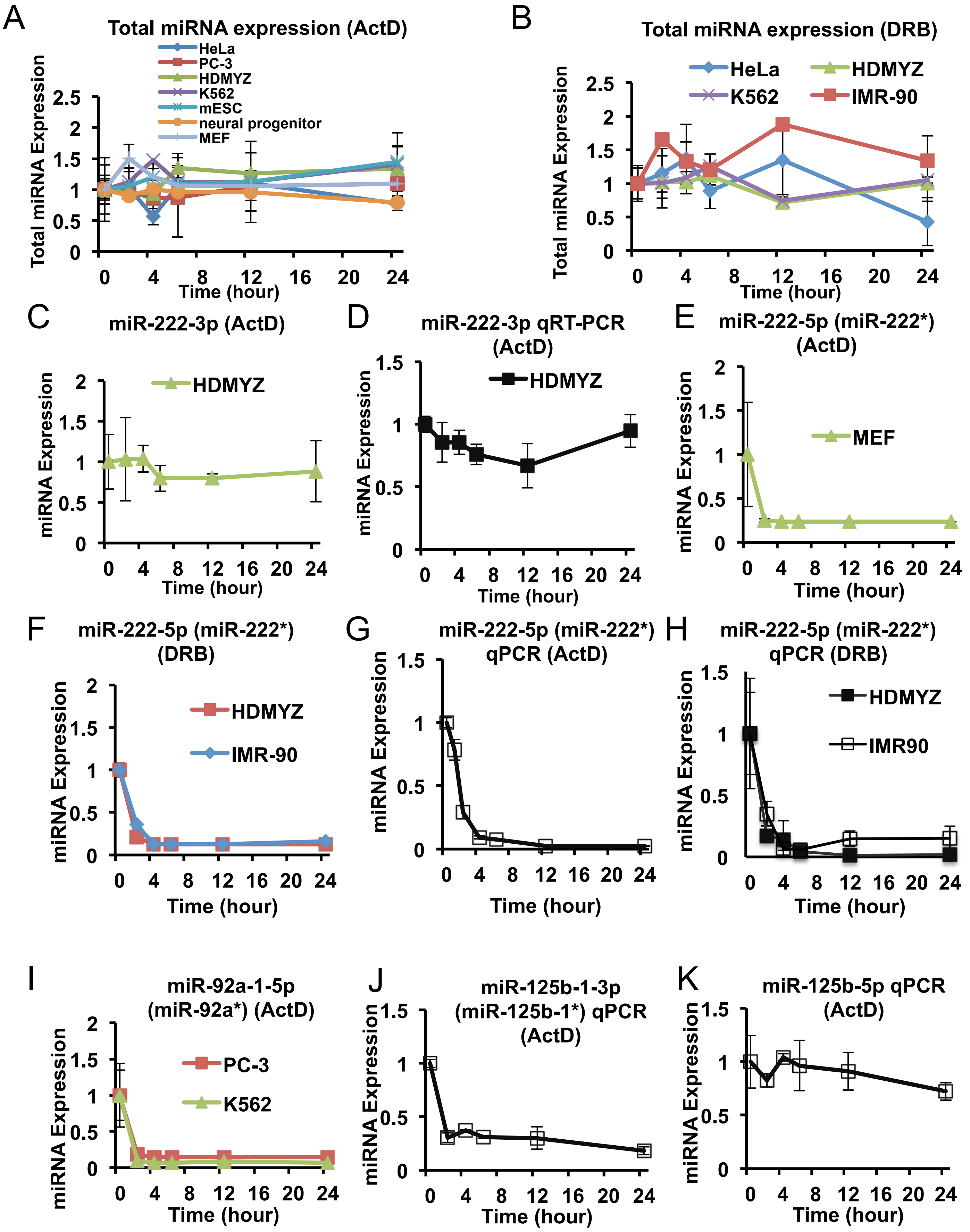

Guo et al. Figure S3

Figure S3. Differential turnover kinetics of miRNAs. (A, B) Total miRNA expression were measured by profiling (see Methods) in the indicated cells after ActD (A) or DRB (B) treatment at indicated time points, and normalized against total RNA amount. **(C, E, F, I)** Levels of miR-222-3p (C), miR-222-5p (E, F) and miR-92a-1-5p (I) measured by miRNA profiling in different cell types at indicated time points post ActD or DRB treatment. For all of the above sub figures, N=2 for ActD treated samples. N=3 for DRB treated samples. **(D, G, H)** miR-222-3p (D) and miR-222-5p (G, H), level measured by Taqman qRT-PCR at indicated time points post ActD or DRB treatment in HDMYZ cells. N=2 for miR-222-3p. N=3 for miR-222-5p. **(J, K)** miR-125b-1-3p (J) and miR-125b-5p (K) level measured by Qiagen qRT-PCR at indicated time points post ActD treatment in HDMYZ cells. N=2. Error bars stand for standard deviations with N=3, or average deviations from median with N=2.

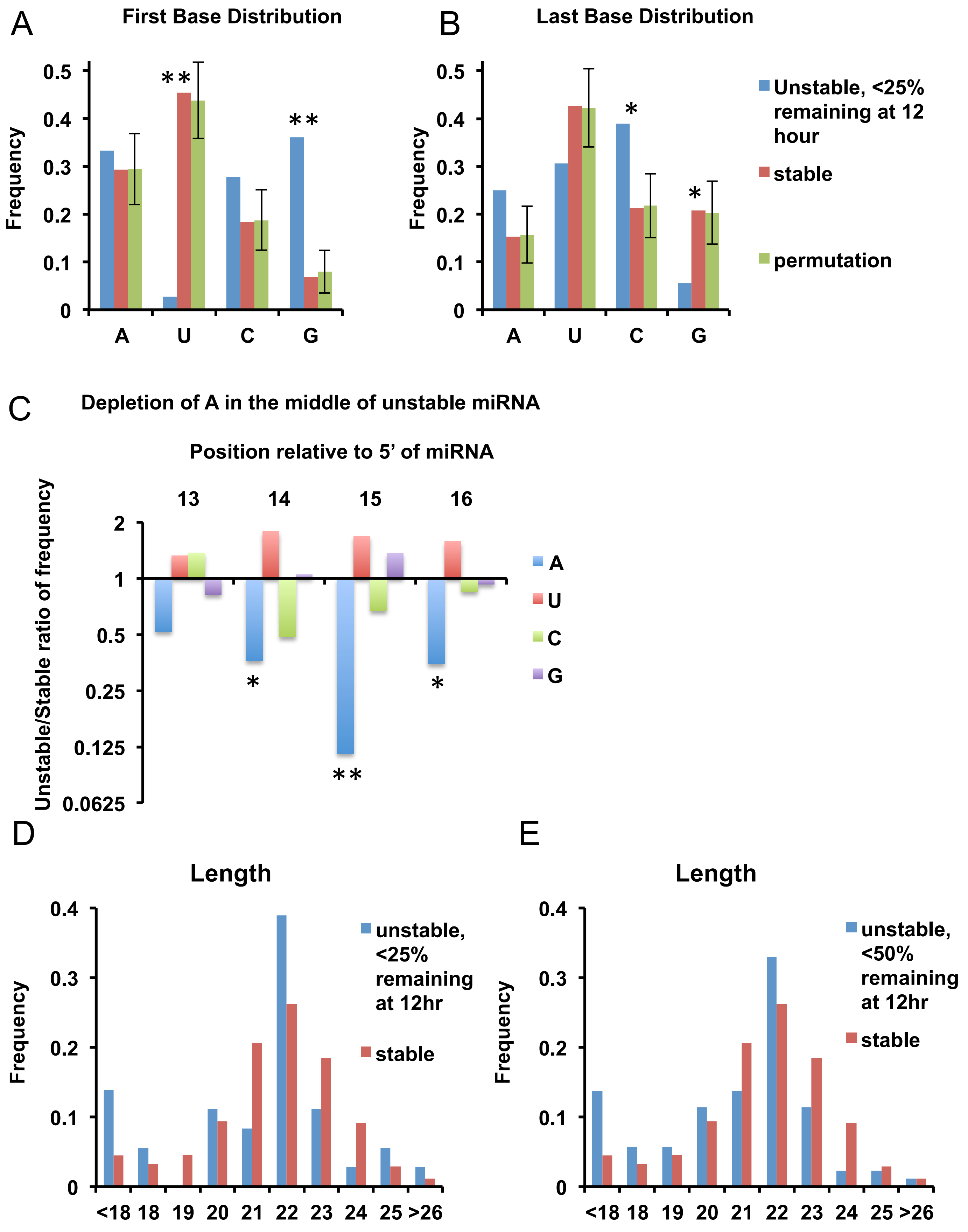

Guo et al. Figure S4

Figure S4. Sequence features of fast turnover miRNAs. (A, B, C) Base-pair features of fast turnover (<25% remaining after 12 hours of ActD treatment with significant miRNA level change) vs. slow turnover (≥75% remaining after 12 hours of ActD treatment) miRNA isoforms. Data collected from 1036 reliably quantifiable miRNA isoform sequences in HDMYZ deep sequencing results. First-base distribution **(A)**, last-base distribution **(B)** and an A-rich motif in the middle of the miRNA **(C)** are shown for miRNA groups with different turnover rates. **(D, E)** Length distribution of fast turnover (<25% (D) or <50% (E) remaining after 12 hours of ActD treatment with significant miRNA level change) vs. slow turnover (see Methods) miRNA isoforms. * $P<0.05$; ** $P<0.01$.

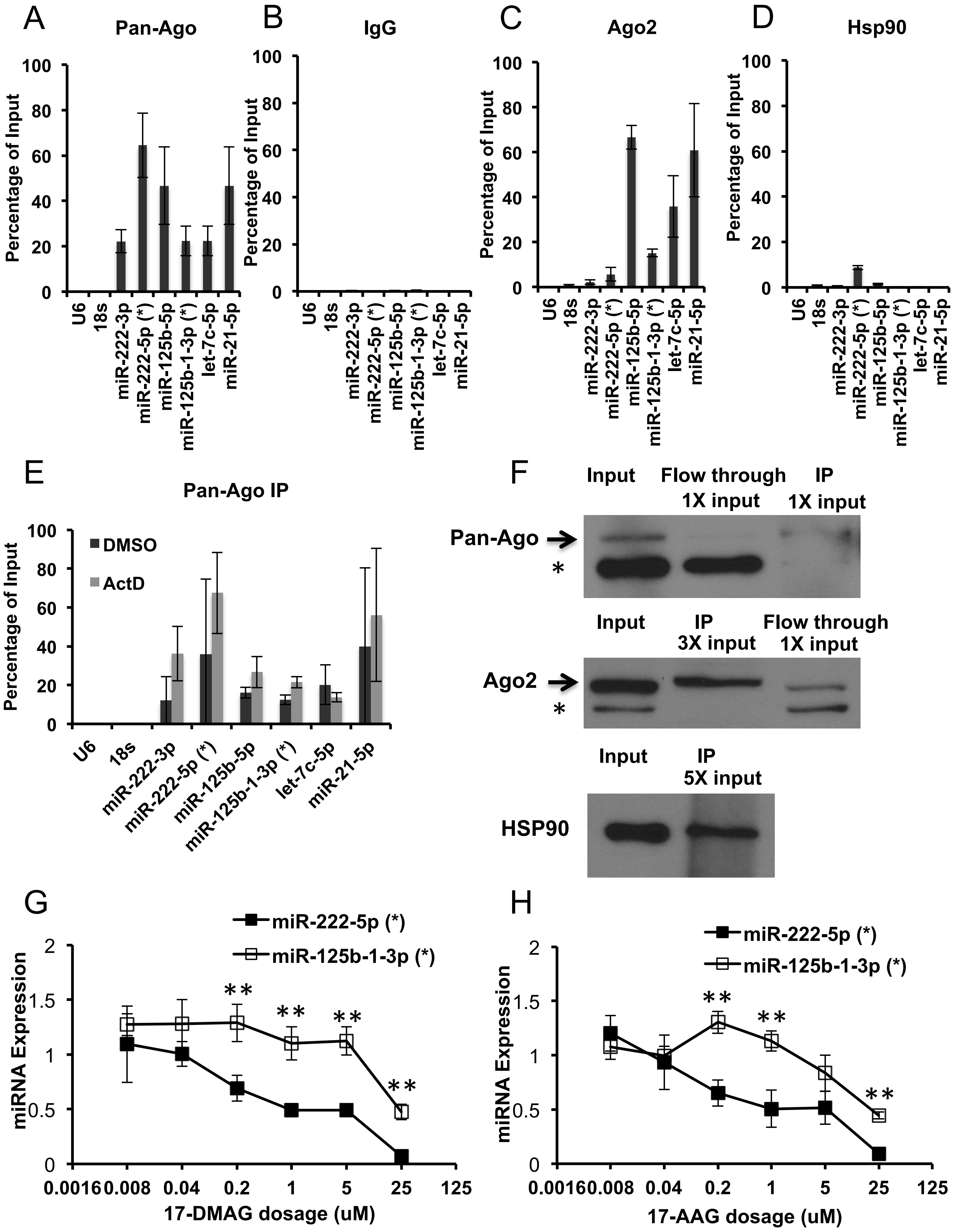

Guo et al. Figure S5

Figure S5. Immunoprecipitation of miRNAs and differential sensitivity toward HSP90 inhibition. (A-E) The same figures as Figure 4A-D and 4G, shown in non-log scale. **(F)** Western blot analyses of Immunoprecipitation samples. Arrows indicate the bands for Pan-Ago or Ago2. Asterisks indicate non-specific bands. Note the preferential depletion of the non-specific bands during IP. **(G, H)** HDMYZ cells were treated with the indicated dosage of 17-DMAG (G) or 17-AAG (H), and harvested after 4 hours. Levels of miR-222-5p (miR-222*) and miR-125b-1-3p (miR-125b-1*) were measured by qRT-PCR. Data were normalized to levels of the corresponding miRNA in HDMYZ cells without drug treatment. N=3. Error bars stand for standard deviation. ** $P<0.01$.

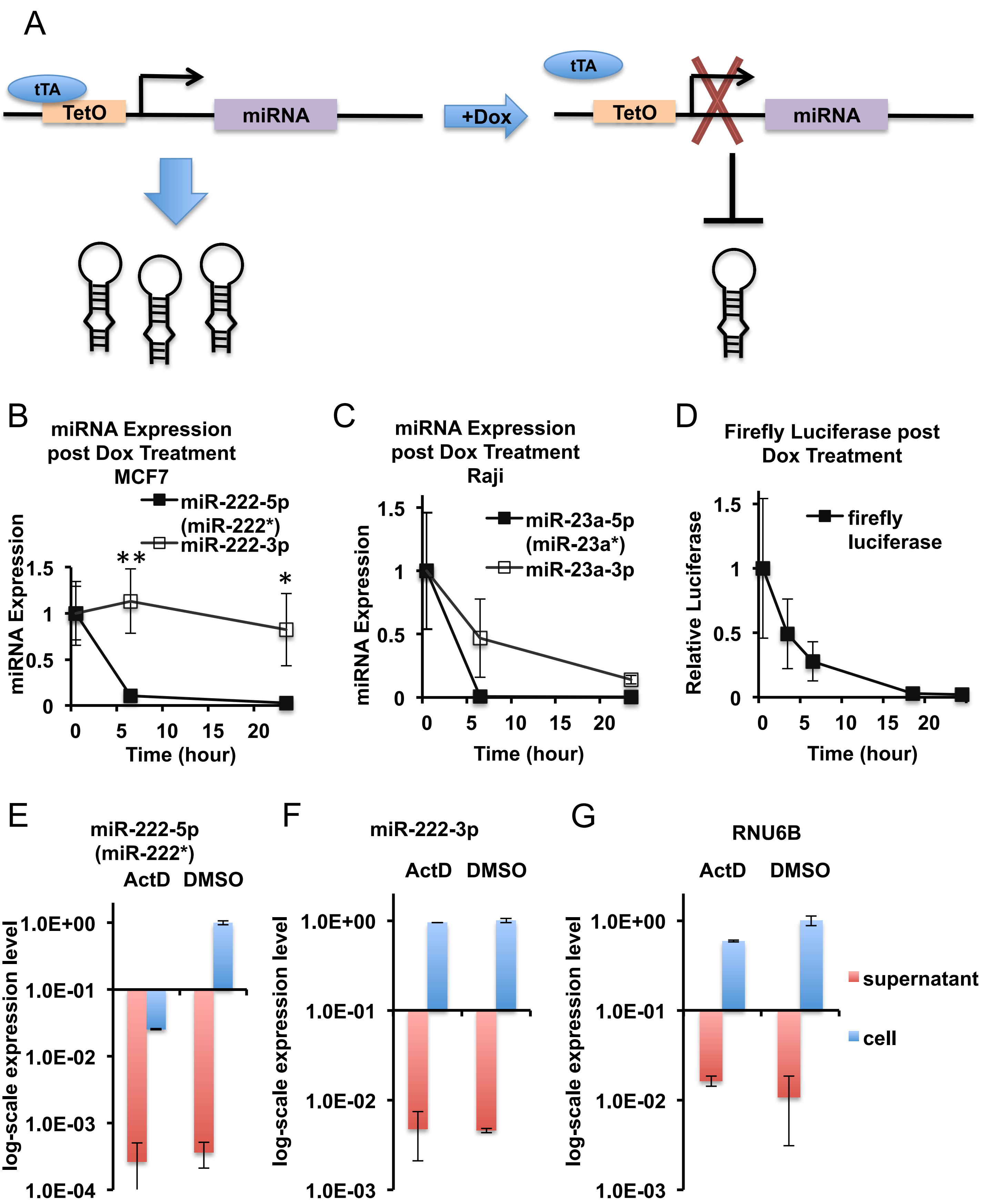

Guo et al. Figure S6

Figure S6. The fast turnover kinetics are intrinsic properties of miRNAs. (A) Schematic of a tet-off inducible system for miRNA expression. Ectopic miRNA expression can be turned off by adding doxycycline (Dox). **(B, C)** miR-222 (B), miR-23a (C) precursors were transduced into MCF-7 (B) or Raji (C) cells, respectively. Corresponding miRNA levels were measured by qRT-PCR (Taqman) after adding Dox to the cells at the indicated time. N=4 for MCF-7 samples. N=2 for Raji samples. Error bars stand for standard deviations with N=4, or average deviations from median with N=2. * $P<0.05$; ** $P<0.01$. **(D)** Turnover kinetics of the firefly luciferase activity post doxycycline treatment in MCF7 cells are shown for the indicated time points. N=3. Error bars stand for standard deviations**. (E-G)** miR-222-5p (E), miR-222-3p (F), RNU6B (G) levels were determined in HDMYZ cells and the corresponding fraction of cell culture medium. Same fractions of cells and cell culture medium from the cell culture were collected for total RNA extraction. RNA was co-precipitated with glycogen. miRNA expression levels were measured with qRT-PCR (Qiagen). N=2. Error bars stand for average deviations from median.

Supplementary References

1. Promega pGL4 Luciferase Reporter Vectors.

2. Guo, S., Bai, H., Megyola, C.M., Halene, S., Krause, D.S., Scadden, D.T. and Lu, J. (2012) Complex oncogene dependence in microRNA-125a-induced myeloproliferative neoplasms. *Proc. Natl. Acad. Sci. U. S. A.*, 10.1073/pnas.1213196109.

3. Lu, J., Guo, S., Ebert, B.L., Zhang, H., Peng, X., Bosco, J., Pretz, J., Schlanger, R., Wang, J.Y., Mak, R.H., et al. (2008) MicroRNA-mediated control of cell fate in megakaryocyte-erythrocyte progenitors. *Dev. Cell*, **14**, 843–53.
